# Not all Group B *Streptococci* are alike: macrophage responses reveal inflammatory and immune-evasive strains

**DOI:** 10.3389/fcimb.2026.1819218

**Published:** 2026-04-16

**Authors:** Larisa Janžič, Lucija Sršen, Sara Petrin, Alojz Ihan, Andreja Nataša Kopitar

**Affiliations:** 1 Laboratory for Cellular Immunology, Institute of Microbiology and Immunology, Faculty of Medicine, University of Ljubljana, Ljubljana, Slovenia; 2 Institute of Pathology, Faculty of Medicine, University of Ljubljana, Ljubljana, Slovenia

**Keywords:** Clinical isolate heterogeneity, Group B *Streptococcus*, immune evasion, inflammation, macrophages, preterm birth, pyroptosis

## Abstract

**Background:**

Group B *Streptococcus* (GBS) remains a leading cause of neonatal sepsis and meningitis despite preventive strategies. Disease severity and clinical presentation vary widely and are influenced by strain-specific virulence traits. Macrophages are key innate immune sentinels during GBS infection; however, how genetically distinct clinical isolates differentially and dynamically reprogram macrophage inflammatory, immunoregulatory, metabolic, and cell death responses remains poorly understood.

**Methods:**

Human THP-1 macrophages were infected with 12 fully characterized clinical GBS isolates representing multiple serotypes, sequence types, clinical presentations, and neonatal gestational ages. Cytokine and chemokine production was quantified at 3 and 24 hours post-infection using LEGENDplex bead-based immunoassays. Caspase-1 activity was measured by bioluminescence, and expression of inflammatory, immunoregulatory, metabolic, and cell death–associated genes were assessed by RT-qPCR at 4 and 24 hours. Data were analyzed using appropriate statistical tests, and multidimensional responses were integrated using radar plot visualization.

**Results:**

Macrophage responses to GBS were highly isolate-specific and varied over time. Serotype Ia and Ib isolates triggered rapid inflammasome-associated activation with early IL-1β and IL-18 release and high caspase-1 activity, consistent with pyroptosis. In contrast, serotype II and especially hypervirulent serotype III isolates showed minimal early inflammasome activation but induced delayed immunoregulatory programs marked by elevated IL-10 and *ACOD1* expression. Reciprocal analyses revealed an inverse relationship between *ACOD1* and IL-1β and a positive association between *ACOD1* and IL-10, indicating coordinated immunometabolic regulation. Serotype-specific glycolytic gene expression signatures appeared at later time points. Stratification by clinical metadata showed that isolates from preterm infants induced stronger early inflammatory responses.

**Conclusions:**

This study shows that GBS pathogenicity is not a uniform species-level trait but reflects isolate-specific abilities to reprogram macrophage immunity. By integrating temporal resolution with strain diversity, it provides a mechanistic framework linking macrophage immune trajectories to preterm birth–associated inflammation and heterogeneous outcomes in neonatal infections.

## 1 Introduction

*Streptococcus agalactiae*, also known as Group B *Streptococcus* (GBS), is a Gram-positive, β-hemolytic commensal colonizing the gastrointestinal and urogenital tracts of healthy individuals but can become pathogenic under certain conditions (Armistead et al., 2019). Although the use of intrapartum antibiotic prophylaxis (IAP) has greatly reduced the incidence of early-onset disease (EOD), GBS remains a leading cause of neonatal sepsis, pneumonia and meningitis and poses a significant health risk not only to newborns but also to pregnant women and immunocompromised adults (Landwehr-Kenzel and Henneke, 2014).

Based on the capsular polysaccharides, GBS can be divided into 10 different serotypes: Ia, Ib, II – IX, with serotypes Ia, Ib, III and V most commonly causing the disease (Pietrocola et al., 2018). Using multi-locus sequence typing (MLST), in which allelic variations in 7 housekeeping genes are analyzed, isolates are further classified into sequence types (ST) and clustered into clonal complexes (CC) based on sequence similarities (Perme et al., 2020). Strains also differ in several virulence factors that determine their pathogenic potential, for example, the amount of β-hemolysin/cytolysin, an ornithine rhamnolipid toxin that promotes host barrier invasion, proinflammatory cytokine responses, erythrocyte lysis, and macrophage inflammatory cell death, pyroptosis (Whidbey et al., 2015). This genetic and phenotypic variability results in a broad spectrum of immune responses, ranging from robust inflammation and rapid cell death to immune evasion and intracellular persistence (Armistead et al., 2019). The unpredictability of these immune interactions contributes to the wide clinical spectrum and often severe nature of GBS infections, which range from asymptomatic colonization to life-threatening sepsis and meningitis (Flaherty et al., 2021).

Despite its clinical importance, the mechanisms underlying the pathogenesis of GBS and its interactions with the host immune system are not yet fully understood. In newborns, whose adaptive immunity is immature, early control of GBS infection relies predominantly on innate immune mechanisms, with macrophages serving as central regulators of antimicrobial defense and inflammatory signaling. Beyond phagocytosis, macrophages coordinate cytokine and chemokine networks that shape downstream immune responses and tissue homeostasis (Galli and Saleh, 2020). During GBS infection, macrophage activation can lead to inflammasome-driven inflammatory responses, including the release of IL-1β and IL-18 and the induction of inflammatory cell death, pyroptosis, through caspase-1 activation. While inflammasome activation is essential for pathogen control, excessive or dysregulated inflammasome signaling has been linked to tissue injury, systemic inflammation, and adverse outcomes in bacterial sepsis (Kumar, 2018).

Experimental evidence suggests that GBS-induced inflammasome activation depends on bacterial virulence factors, particularly β-hemolysin/cytolysin, which facilitates inflammasome assembly and downstream inflammatory signaling (Whidbey et al., 2015; Costa et al., 2012). However, existing studies remain limited in scope, typically focusing on one or two laboratory strains or serotypes and often assessing immune responses at a single, late time point. This narrow experimental design restricts our understanding of how strain-specific diversity and early immune dynamics influence inflammasome activation, metabolic adaptation, and disease outcome. In parallel, macrophage metabolism is increasingly recognized as a critical regulator of inflammatory signaling, yet how genetically distinct GBS isolates differentially reprogram macrophage immunometabolism remains largely unexplored.

Most previous *in vitro* studies therefore fail to capture early, strain-specific immune events that may critically shape downstream inflammation and clinical progression. To address this gap, we applied a time-resolved experimental approach using fully genotyped and phenotyped clinical GBS isolates to systematically compare human macrophage responses across early and late stages of infection. By integrating cytokine profiling, gene expression analysis, caspase-1 activity, and detailed clinical metadata – including serotype, sequence type, specimen origin, and neonatal characteristics – this study aimed to define isolate-specific immune response patterns. We hypothesized that genetically and clinically distinct GBS isolates differentially reprogram macrophage immune responses over time, resulting in divergent inflammatory and immunoregulatory trajectories characterized by early inflammasome-driven pyroptosis or delayed immunometabolic immune evasion. These findings build upon our previous work characterizing macrophage phagocytosis, surface marker expression, and immunometabolic remodeling, and together provide a unified framework for understanding how distinct GBS lineages reprogram macrophage function over time (Janzic et al., 2023).

Here, we show that clinical isolates of Group B *Streptococcus* elicit qualitatively distinct innate immune programs in macrophages, ranging from early inflammasome-driven pyroptotic cell death to delayed immunosuppressive and metabolically adapted activation states. Importantly, GBS pathogenicity is not a uniform species-level trait but is driven by isolate-specific capacities to reprogram macrophage immunity. Isolates associated with preterm birth and invasive neonatal disease preferentially skew responses toward pathological inflammation or immune dysfunction, with direct implications for neonatal sepsis.

## 2 Materials and methods

### 2.1 THP-1 cell line

The acute monocytic human leukemia THP-1 cell line (ATCC^®^ TIB-202™) was cultured in RPMI 1640 medium, supplemented with 2 mM L-glutamine, 25 mM D-glucose, 1 mM sodium pyruvate, 10 mM HEPES (Gibco, Thermo Fisher Scientific) and 10% fetal bovine serum (Sigma-Aldrich) at 37 °C with 5% CO_2_. The cells were passaged every 3–4 days.

### 2.2 Macrophage differentiation

Prior to bacterial infections, THP-1 monocytes were differentiated into macrophages by incubation with 100 nM phorbol 12-myristate 13-acetate (PMA; Sigma-Aldrich) for 3 days, followed by 5-day rest in a medium without PMA. The differentiation protocol was selected based on preliminary experiments, in which different PMA concentrations (30, 100, and 162 nM) were used for different periods of time (24h and 1-day rest, 72h and 1-day rest and 24 or 72h and 5-day rest), as previously described (Janzic et al., 2023).

### 2.3 Bacterial isolates

Twelve different GBS isolates, representing different serotypes, STs, and CCs, were selected (**Table 1**). The isolates vary in clinical presentation (invasive or colonizing) and were obtained from various specimens (blood, cerebrospinal fluid, or vagina/vagina-rectum), either from infected newborns of different gestational ages or from colonized pregnant women. All isolates were previously genotyped by Perme et al. (2020) (Perme et al., 2020) and are kept at the Institute of Microbiology and Immunology, Faculty of Medicine, University of Ljubljana. The characteristics of the GBS isolates used in our study are summarized in **Table 1**.

**Table 1:** Characteristics of selected GBS isolates and related clinical data from affected neonates.

Isolate ID	Serotype	Sequence type (ST)	Clonal complex (CC)	Clinical presentation	Specimen	Gestational age of the newborn	Age of the newborn at the time of sampling
229	Ia	ST-23	CC-23	LOD	CSF	30 weeks*	48 days
9427		ST-144	CC-23	Colonization	V/V-R		
203	Ib	ST-8	CC-12	EOD	Blood	31 weeks*	1 day
10276		ST-9	CC-1	Colonization	V/V-R		
211	II	ST-28	CC-19	EOD	Blood	40 weeks***	1 day
7339		ST-12	CC-12	Colonization	V/V-R		
231	III	ST-17	CC-17	EOD	Blood	39 weeks***	2 days
9731		CT-17	CC-17	Colonization	V/V-R		
8422	IV	ST-291	CC-17	Colonization	V/V-R		
6	V	ST-1	CC-1	EOD	CSF	38 weeks***	0 days
104		ST-1	CC-1	EOD	Blood	35 weeks**	0 days
123		ST-498	CC-498	EOD	Blood	34 weeks**	1 days

*A gestational age of 28 to <32 weeks indicates a very preterm (VP) newborn; **a gestational age of 32 to <37 weeks indicates a moderate or late preterm (MP) newborn; ***a gestational age of >37 weeks indicates a term born (TB) newborn (Quinn et al., 2016). LOD, Late onset disease; EOD, Early onset disease; CSF, cerebrospinal fluid; V/V-R, vagina/vagina-rectum.

Bacteria were grown from frozen stocks on blood agar plates in ambient air at 37 °C overnight. The following day, a single bacterial colony was sub-cultured from blood agar plates in Todd-Hewitt broth (THB) and incubated under aerobic shaking conditions at 37 °C in ambient air, overnight.

### 2.4 Bacterial infection of THP-1 macrophages

Prior to bacterial infection, GBS isolates were harvested by centrifugation, washed in sterile phosphate-buffered saline (PBS), and opsonized in human serum at 37 °C for 30 min, with shaking. Human serum used as a source of complement for bacterial opsonization was obtained from blood donated voluntarily by healthy staff members from our department. Bacteria were washed again and resuspended in antibiotic-free cell culture medium. Bacterial density was measured using the DensiCHEK Plus Instrument (BioMerieux) optical densitometer and was set to OD_580_ = 0.5, which corresponds to a concentration of 1.5 x 10^8^ CFU/ml. The accuracy of the densitometer was regularly checked by appropriate serial dilutions and CFU quantification.

Differentiated macrophages were washed in PBS and then GBS isolates were added with a multiplicity of infection (MOI) of 10 bacteria per macrophage and incubated at 37 °C and 5% CO_2_ for 3 hours, unless otherwise noted.

### 2.5 Cytokine detection with the LEGENDplex™ bead-based immunoassay

To measure cytokine production and secretion by unstimulated and GBS-infected macrophages, 4 x 10^5^ cells/ml were seeded in a 12-well plate and differentiated into macrophages for 8 days as described in section 2.2. To gain insight into the dynamics of cytokine production, supernatants of infected macrophages were collected at two time points – 3 and 24 hours after the onset of infection. For this purpose, 2 plates of macrophages were differentiated and infected in each experiment, one for collecting supernatants 3 hours after infection and the other for collecting supernatants 24 hours after infection. Macrophages were infected with individual GBS isolates for 3 hours, as described in section 2.4. Unstimulated macrophages served as the negative control. After incubation, supernatants for cytokine production measurement at 3 hours post-infection were transferred from the plate to centrifuge tubes, centrifuged to remove all cells and bacteria, and stored at -70 °C until further analysis. Supernatants collected after 3 hours corresponded to the end of the infection period and were obtained immediately before antibiotic treatment to quantify cytokines released during bacterial exposure. To determine cytokine production at 24 hours after infection, extracellular bacteria were removed by washing away unattached bacteria and adding fresh RPMI 1640 supplemented with 2% FBS, 100 µg/ml gentamicin (Krka), and 5 µg/ml penicillin G (Sandoz). Cells were then incubated for an additional 21 hours, after which supernatants were collected and stored at -70 °C until further analysis.

To quantify multiple cytokines, the supernatants were analyzed using a bead-based custom made LEGENDplex immunoassay (BioLegend). The following human cytokines and chemokines were measured: IL-1β, IL-6, IL-10, IL-12p40, IL-18, TNF-α, CCL8 (MCP2), CXCL8 (IL-8), and CXCL9 (MIG). All experiments were performed according to the manufacturer’s instructions. Cytokines were measured with a BD FACSCanto II flow cytometer (Becton Dickinson, BD), and the data were analyzed using LEGENDplex™ Data Analysis Software (BioLegend).

### 2.6 Caspase-1 activity assay

Caspase-1 activity was determined in the supernatants of infected macrophages using the commercially available Caspase-Glo^®^ 1 Inflammasome Assay (Promega) according to the manufacturer’s instructions. Briefly, THP-1 monocytes were seeded in a 96-well plate at a density of 4 x 10^5^ cells/ml, differentiated into macrophages as described in section 2.2, and infected with 100 µl aliquots of bacterial suspensions. After 3 hours, cell supernatants were collected in a new plate, and either Caspase-Glo 1 reagent (containing the MG-132 proteasome inhibitor) or Caspase-Glo 1 YVAD-CHO reagent (containing ac-YVAD-CHO, a selective caspase-1 inhibitor) was added to individual wells. Plates were gently mixed on a plate shaker at 400 rpm for 30 seconds and then incubated at room temperature for 2 hours to allow stabilization of the bioluminescent signal. Bioluminescence was measured using the Cytation 5 Multi-Mode Reader (BioTek).

### 2.7 Gene expression analysis

To analyze the expression of nine different genes involved in glucose metabolism, inflammation, and cell death, real-time quantitative PCR (qPCR) assays were performed. Because RNA was isolated at two time points, 4 and 24 hours after the onset of infection, two plates of macrophages were infected in each experiment. Differentiated THP-1 macrophages, seeded in a 6-well plate at a density of 4 x 10^5^ cells/ml, were left untreated or treated with GBS isolates as described in section 2.4, or with 400 ng/ml LPS (Sigma-Aldrich) as a positive control. After 3 hours, the culture medium was aspirated from each well, and fresh RPMI 1640 supplemented with 2% FBS, 100 µg/ml gentamicin, and 5 µg/ml penicillin G was added for an additional 1 or 21 hours, at which point RNA was isolated. The time points were selected based on preliminary optimization experiments using one representative GBS isolate, in which several time points (1, 3, 4, 18, and 24 hours) were tested, revealing that transcriptional differences were most clearly detectable after 4 and 24 hours of infection.

#### 2.7.1 RNA isolation and reverse transcription

Total RNA was isolated using the RNeasy^®^ Plus Mini Kit (Qiagen). Briefly, after 4 or 24 hours of incubation, the culture medium with antibiotics was removed, and cells were lysed by adding 600 µl of lysis buffer and scraping. Lysed cells were transferred to a gDNA eliminator spin column to remove genomic DNA. Ethanol was added to the flow-through to provide appropriate binding conditions for RNA, and samples were applied to RNeasy spin column. Contaminants were washed away, and RNA was eluted from the column by adding RNase-free water (Qiagen). RNA concentration was quantified with a Qubit 4 Fluorometer (Thermo Fisher Scientific) and a Qubit^®^ RNA Broad-range Assay Kit (Thermo Fisher Scientific). Reverse transcription was performed on 0.2 µg of total RNA using the High-Capacity cDNA Reverse Transcription Kit (Applied Biosystems) according to the manufacturer’s instructions. The resulting cDNA was stored at -70 °C until further use.

#### 2.7.2 Real-time quantitative PCR

To determine gene expression, qPCR analyses were performed using TaqMan Fast Advanced Master Mix (Applied Biosystems) according to the manufacturer’s instructions, with TaqMan Assay probes (Applied Biosystems) specific for *PFKFB3* (Assay ID: Hs00998698_m1), *SLC2A1* (Assay ID: Hs00892681_m1), *HIF1A* (Assay ID: Hs00153153_m1), *IL1B* (Assay ID: Hs01555410_m1), *IL10* (Assay ID: Hs00961622_m1), *ACOD1* (Assay ID: Hs00985781_m1), *CASP1* (Assay ID: Hs00354836_m1) and *CASP3* (Assay ID: Hs00234387_m1), respectively. *RPL37A* (Assay ID: Hs01102345_m1) was used as an endogenous control.

### 2.8 Statistical analysis

All data were tested for normality using the Shapiro–Wilk or Kolmogorov–Smirnov test. Depending on the data distribution, unpaired t-tests or one-way ANOVA with *post hoc* Tukey’s or Šidák’s multiple-comparisons tests (for normally distributed data), and Mann–Whitney or Kruskal–Wallis tests with *post hoc* Dunn’s multiple-comparisons tests (for nonparametric data) were used to assess statistical significance. A p value ≤ 0.05 was considered statistically significant. Statistical analyses were performed using GraphPad Prism software (version 10.0, GraphPad Software Inc.), and all graphs were generated using GraphPad Prism. For radar plot analyses, selected immune, metabolic, and cell death–associated parameters were processed to enable direct comparison across variables measured on different scales. For each parameter, values were normalized across all isolates using min–max scaling within each parameter, with normalized values ranging from 0 to 1. The same scaling was applied consistently across all serotypes to preserve relative differences between serotypes and prevent any single parameter from dominating the visualization due to differences in measurement units or dynamic range. Normalized values were then aggregated at the serotype level by calculating mean responses for isolates belonging to the same serotype. Radar plots were used as an integrative visualization tool to summarize multidimensional response patterns across serotypes, rather than for statistical inference or hypothesis testing. All radar plots were generated using Canva (Canva Pty Ltd.).

## 3 Results

### 3.1 Isolate-specific induction of chemokine responses in macrophages over time

**Figure 1:**
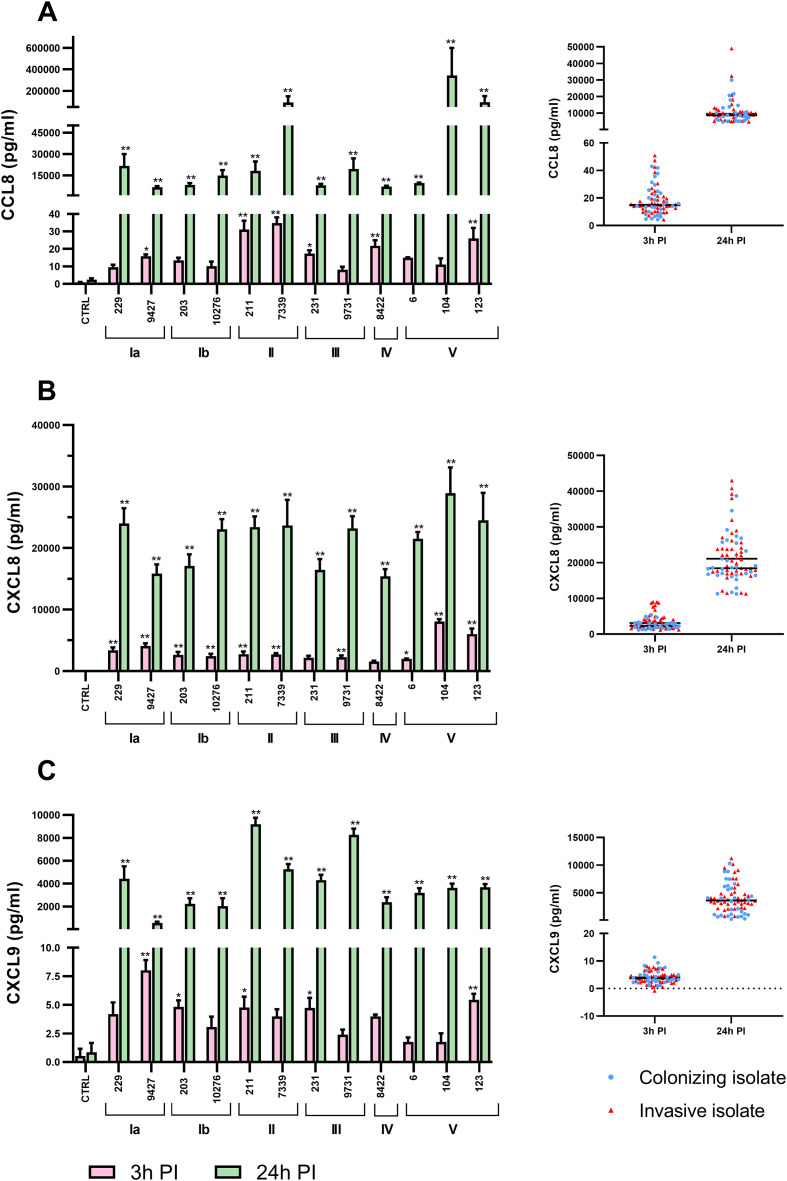
Temporal dynamics of chemokine production following macrophage infection with 12 different GBS isolates. THP-1 macrophages were infected with GBS isolates at a MOI of 10 for 3 hours or 24 hours, after which supernatants were collected, and chemokine production was determined using bead-based LEGENDplex immunoassay. The bar graphs and dot plots show the concentrations of the chemokines **(A)** CCL8 (MCP-2), **(B)** CXCL8 (IL-8), and **(C)** CXCL9 (MIG) at two different time points (left) or according to clinical presentation of GBS isolates (colonizing or invasive, right), respectively. Data from three independent biological replicates, each with two technical replicates, are shown as mean ± SEM (n = 6). Asterisks above the bar graphs (*p < 0.05 and **p ≤ 0.01) indicate statistically significant differences compared to unstimulated control macrophages, as determined by one-way ANOVA and *post-hoc* Šidák’s multiple comparison test. Statistically significant differences between macrophages stimulated with different isolates are shown in **Supplementary Tables S1**, **S11–S14**. PI, post-infection.

Infection of THP-1 macrophages with different GBS isolates resulted in markedly different chemokine responses, both in magnitude and kinetics, highlighting strain-specific modulation of the innate immune response. Quantification of three chemokines involved in immune cell recruitment revealed significantly increased levels 24 hours post-infection compared to unstimulated controls (**Figure 1**).

CCL8 (MCP-2) production peaked 3 hours post-infection in macrophages stimulated with serotype II isolates (211, 7339), followed by serotype IV (8422) and V (123) isolates, reaching concentrations up to 40 pg/mL. After 24 hours, certain serotype V isolates (104, 123) induced CCL8 secretion exceeding 400.000 pg/mL, while in other macrophages concentrations ranged between 15.000 and 30.000 pg/mL (**Figure 1A**). Similarly, CXCL8 levels were highest 3 hours post-infection in response to serotype V isolates (104, 123) (up to 800 pg/mL) and increased significantly by 24 hours post-infection (**Figure 1B**). In contrast, CXCL9 showed a unique temporal profile: peak levels were observed 3 hours post-infection in response to the colonizing serotype Ia isolate (9427), while after 24 hours, CXCL9 production in these macrophages decreased to the lowest levels among all isolates tested (**Figure 1C**).

The kinetics of chemokine induction varied considerably not only between GBS isolates but also between individual chemokines. CXCL8 showed an early and robust response, with concentrations between 100 and 900 pg/mL 3 hours post-infection. In contrast, CCL8 production was delayed but increased dramatically, reaching peak levels (over 200.000 pg/mL) only after 24 hours. However, no differences were observed between macrophages infected with invasive or colonizing isolates (**Figure 1**, dot plots on the right). Complete statistical analyses of the chemokine production profiles of macrophages infected with individual isolates are provided in **Supplementary Tables S1**, **S2**, **S11–S14**.

### 3.2 Isolate -specific and time-dependent inflammatory responses in macrophages following GBS infection

**Figure 2:**
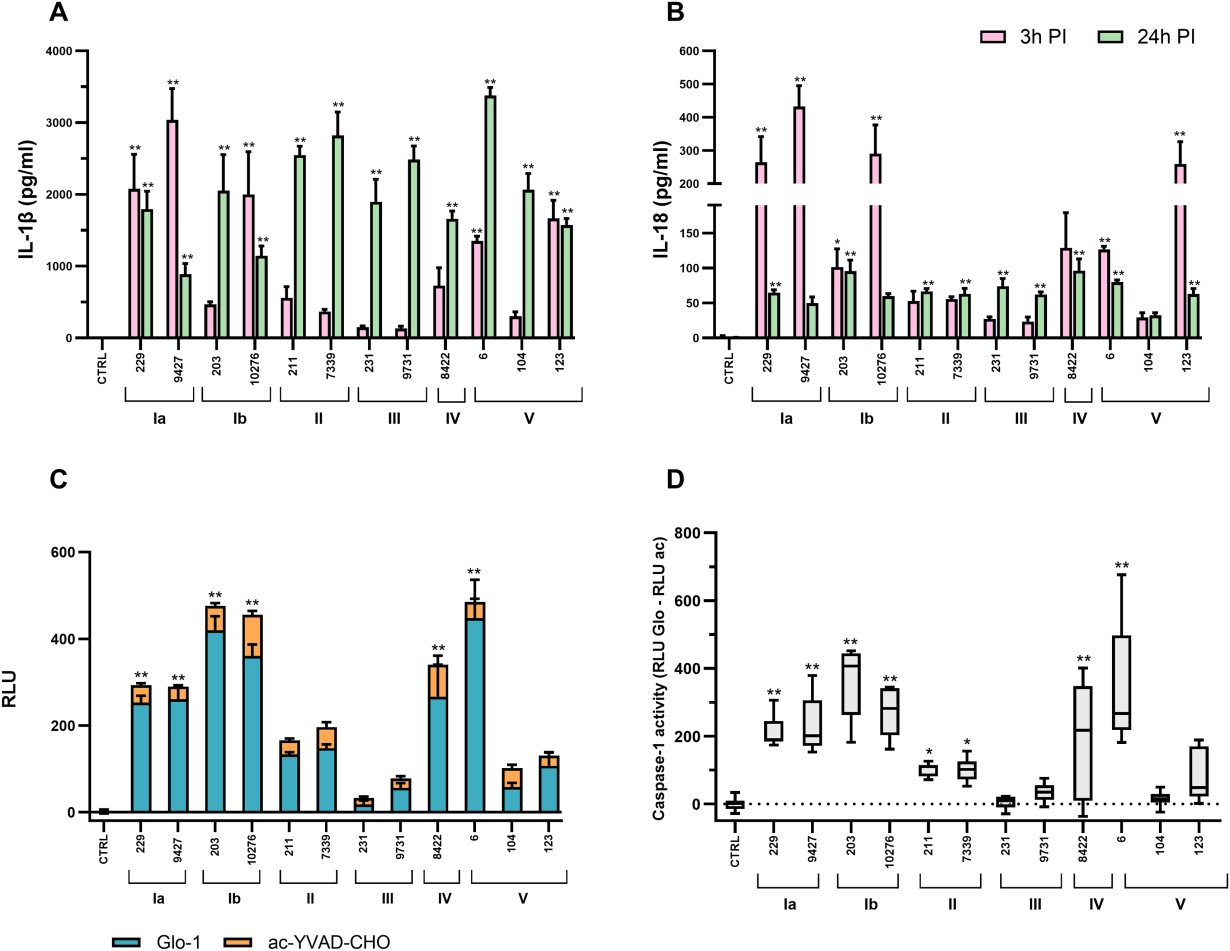
Isolate-dependent inflammation and caspase-1 activity in THP-1 macrophages infected with GBS isolates. The release of inflammatory cytokines **(A)** IL-1β and **(B)** IL-18 was measured in cell supernatants at 3 hours (pink) and 24 hours (green) after infection with 12 different GBS isolates. Data from three independent biological replicates, each with two technical replicates, are presented as mean ± SEM (n = 6). **(C)** Total caspase activity (Glo-1, shown in blue) and caspase activity after addition of the caspase-1-selective inhibitor ac-YVAD-CHO (shown in orange) were determined 3 hours after infection using a bioluminescence assay. **(D)** The boxplots show the specific activity of caspase-1, determined by subtracting the activity after addition of the inhibitor ac-YVAD-CHO from the total caspase activity. All experiments were performed in three independent biological replicates with three technical replicates each (n = 9), shown as mean values ± SEM. Asterisks above the graphs indicate statistically significant differences compared to unstimulated macrophages, where *p ≤ 0.05 and **p ≤ 0.01, as determined by one-way ANOVA and *post-hoc* Šidák’s multiple comparisons test, respectively. Statistically significant differences between macrophages stimulated with individual isolates are summarized in **Supplementary Tables S3**, **S4**, **S15** and **S16** for clarity. PI, post-infection; RLU, relative light units.

To characterize inflammatory responses induced by distinct GBS isolates, the concentrations of the pro-inflammatory cytokines IL-1β and IL-18 were measured in macrophage supernatants, and caspase-1 activity was assessed. Together, these parameters are widely used indicators of NLRP3 inflammasome activation and inflammatory cell death, i.e., pyroptosis. Significant isolate- and time-dependent differences were observed across all readouts, indicating substantial heterogeneity in macrophage inflammatory responses to GBS (**Figure 2**).

At the early time point (3 h post-infection), macrophages infected with serotype Ia isolates (9427, 229), a colonizing serotype Ib isolate (10276), and selected serotype V isolates (6, 123) exhibited the strongest inflammatory responses, characterized by high concentrations of both IL-1β and IL-18 (**Figures 2A, B**). IL-1β levels reached approximately 4.000 pg/mL, while IL-18 concentrations peaked at around 500 pg/mL. In contrast, macrophages exposed to serotype III isolates (231, 9731) and one serotype V isolate (104) produced significantly lower levels of both cytokines at this early time point, indicating attenuated early inflammatory signaling. At 24 h post-infection, IL-1β production increased across all serotypes, including those that elicited weak early responses, suggesting delayed induction of IL-1β–associated inflammation in a subset of isolates (**Figure 2A**). In contrast, IL-18 production did not differ significantly between time points, except for serotype Ia and Ib isolates, where IL-18 levels decreased markedly at 24 h post-infection (**Figure 2B**).

Cytokine production closely mirrored caspase-1 activity assessed using a bioluminescence-based assay. The highest caspase-1 activity was observed in macrophages infected with serotype V isolate (6), followed by serotype Ib, Ia, and IV isolates (**Figure 2C**). In contrast, serotype III and II isolates induced little or no significant caspase-1 activity. Except for serotype V, which displayed pronounced intra-serotype variability, isolates belonging to the same serotype generally elicited comparable caspase-1 activity profiles. To assess whether the measured caspase activity reflected caspase-1–associated signaling, macrophages were treated with the selective caspase-1 inhibitor ac-YVAD-CHO. In most conditions, caspase activity was strongly reduced in the presence of the inhibitor, indicating that the detected signal predominantly reflected caspase-1–associated activity. In contrast, macrophages infected with serotype III and II isolates showed limited inhibition, suggesting that residual caspase activity under these conditions may involve additional proteases.

Overall, serotypes Ia, Ib, and selected serotype V isolates induced strong early inflammatory responses characterized by high IL-1β and IL-18 production together with elevated caspase-1 activity, whereas serotype III and II isolates consistently elicited weaker early cytokine responses and minimal caspase-1 activation. These data demonstrate that macrophage inflammatory responses to GBS are highly serotype-dependent and dynamically regulated over time.

### 3.3 IL-1β and IL-18 production is associated with caspase-1 activity, macrophage lysis, and gestational age of the neonates

**Figure 3:**
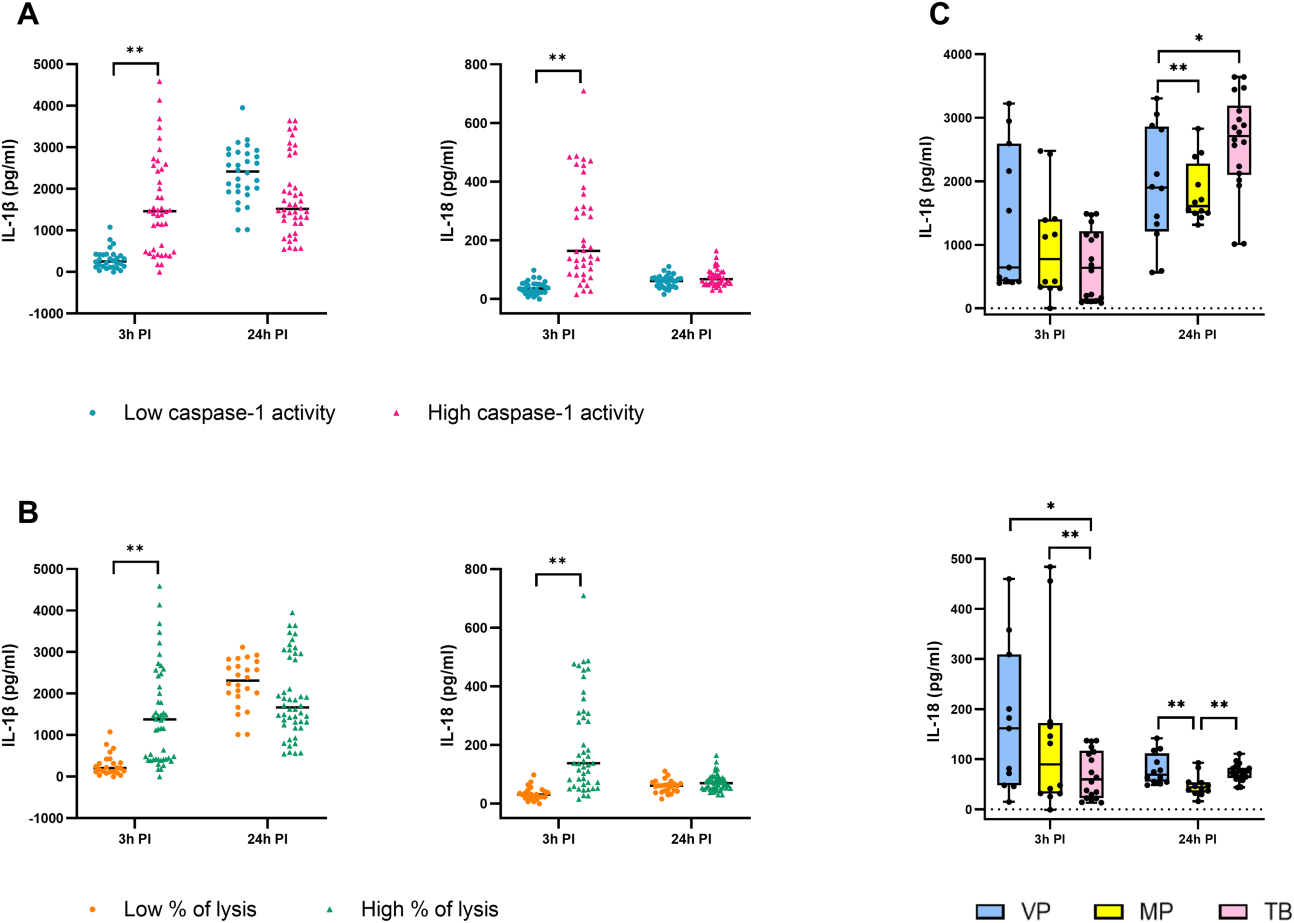
Inflammatory cytokines in relation to caspase-1 activity, macrophage lysis, and gestational age of the neonates. The graphs show IL-1β and IL-18 production according to **(A)** caspase-1 activity, **(B)** macrophage lysis, and **(C)** gestational age of the newborns (VP, severely/very preterm, 28 to <32 weeks; MP - moderately preterm, 32 to <37 weeks; TB, term born, over 37 weeks). Data from three independent biological replicates, each with two technical replicates, are presented (n = 6). *p ≤ 0.05, **p ≤ 0.01, determined by Kruskal-Wallis test and Dunn’s multiple comparison test, or by unpaired t-test or Mann-Whitney test. PI, post-infection.

To further define the relationship between inflammatory cytokine production and inflammatory cell death, IL-1β and IL-18 levels were analyzed in relation to caspase-1 activity and macrophage lysis, measured by LDH release in our previous study (Janzic et al., 2023), as well as the gestational age of the neonates from whom the invasive GBS isolates were obtained (**Figure 3**).

Three hours post-infection, macrophages with high caspase-1 activity released significantly greater amounts of IL-1β and IL-18 compared to macrophages with low caspase-1 activity (**Figures 3A, C**). A similar pattern was observed when cytokine production was stratified by macrophage lysis - isolates that induced high LDH release were associated with markedly increased IL-1β and IL-18 secretion relative to isolates inducing low levels of lysis (**Figures 3B, D**). For both stratifications, cytokine concentrations were approximately fourfold higher in the high caspase-1 activity or high lysis groups, indicating a close association between inflammatory cytokine release, caspase-1 activation, and membrane damage early after infection. This early increase in cytokine secretion supports the coupling of NLRP3 inflammasome activation with the release of inflammatory cytokines in response to certain GBS isolates. At 24 hours post-infection, differences in IL-1β and IL-18 production between high and low caspase-1 activity or lysis groups were substantially reduced, suggesting a temporal convergence of cytokine responses independent of the magnitude of early inflammatory cell death (**Figures 3A–D**).

Cytokine production was further analyzed in relation to the gestational age of the neonates. Macrophages infected with GBS isolates from very preterm (VP) and moderate/late preterm (MP) infants produced the highest concentrations of IL-1β and IL-18 at 3 hours post-infection (**Figures 3E, F**). Although IL-1β levels showed a consistent trend toward higher production in response to isolates from VP infants compared with MP and term-born (TB) infants, these differences did not reach statistical significance (**Figure 3E**). In contrast, IL-18 production was significantly higher in macrophages infected with isolates from VP and MP infants compared with those infected with isolates from TB infants (**Figure 3F**).

Together, these results show that early IL-1β and IL-18 release is closely linked to caspase-1 activity and macrophage lysis and is more pronounced in response to GBS isolates from preterm neonates, suggesting a possible link to increased inflammation and preterm birth. The coordinated induction of caspase-1 activity, inflammatory cytokine release, and cell lysis is consistent with the involvement and activation of the NLRP3 inflammasome following infection with specific GBS isolates. This association is most evident at early time points after infection and decreases over time, highlighting the dynamic nature of NLRP3-associated inflammatory responses during GBS–macrophage interactions.

### 3.4 Serotype- and gestational age-dependent modulation of pro- and anti-inflammatory cytokine responses during GBS infection

**Figure 4:**
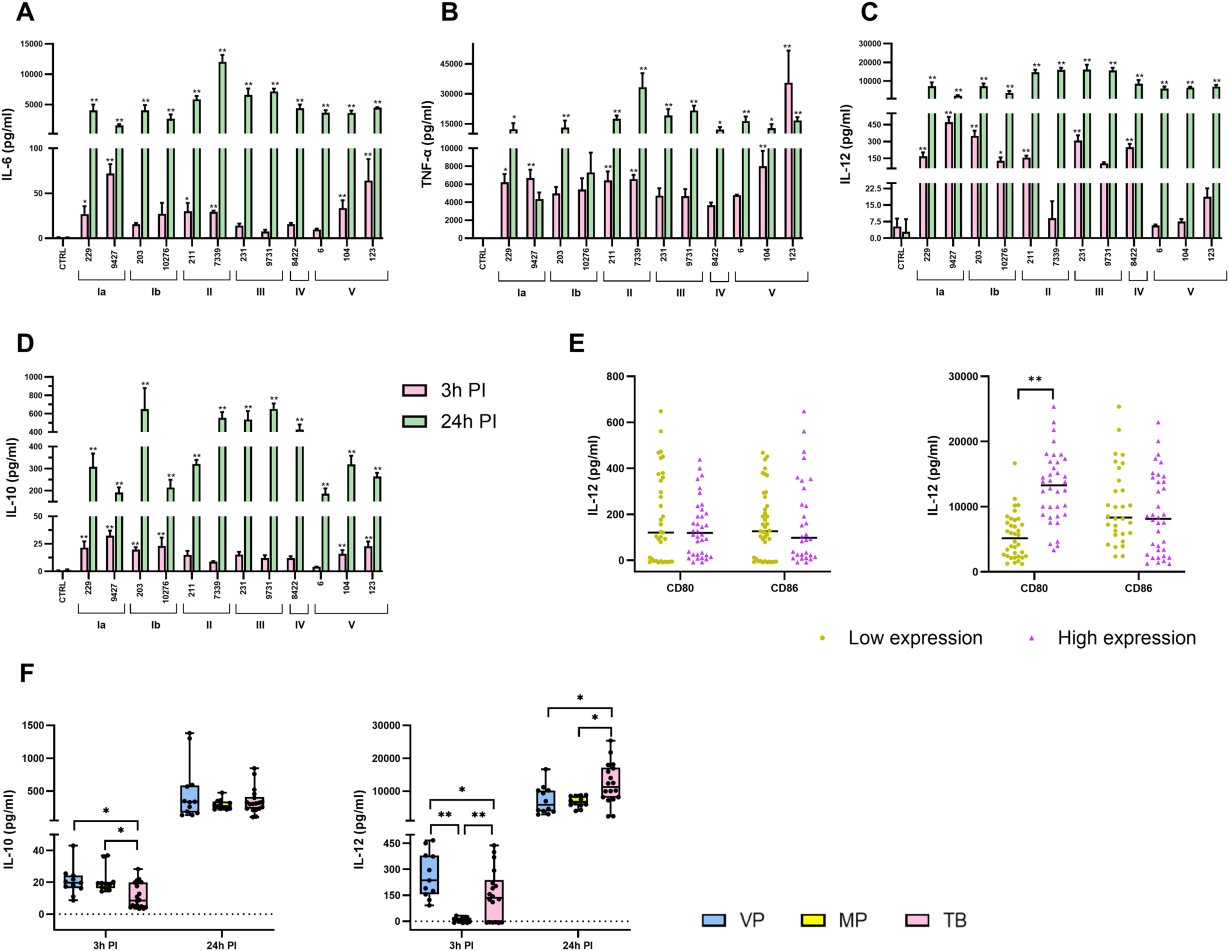
Production of inflammatory and anti-inflammatory cytokines in relation to serotype, co-stimulatory molecules, and gestational age. The bar graphs show the concentrations of the inflammatory cytokines **(A)** IL-6, **(B)** IL-12, **(C)** TNF-α, and the anti-inflammatory cytokine **(D)** IL-10 measured in the supernatants of infected macrophages at 3 hours (pink) and 24 hours (green) after infection. The graphs show mean values ± SEM for three independent biological replicates, each with two technical replicates (n = 6). Asterisks above the graphs indicate statistically significant differences from unstimulated macrophages, while statistically significant differences between macrophages stimulated with different isolates are shown in **Supplementary Tables S5–S10** and **S17**, S18 for clarification. **(E)** Dot plots show the production of IL-12 according to the expression of two co-stimulatory molecules, CD80 and CD86, at 3 hours (left) and 24 hours (right) after infection. **(F)** The concentrations of IL-10 (left) and IL-12 (right) according to the gestational age of the neonates (VP, severely/very preterm [28 to <32 weeks]; MP, moderately preterm [32 to <37 weeks]; TB, term born [over 37 weeks]). *p ≤ 0.05 and **p ≤ 0.01, determined by one-way ANOVA and *post-hoc* Šidák multiple comparison test, Kruskal-Wallis test with Dunn’s multiple comparison test, unpaired t-test, or Mann-Whitney test, respectively. PI, post-infection.

To characterize macrophage cytokine responses beyond inflammasome-associated mediators, the concentrations of the pro-inflammatory cytokines IL-6, IL-12, and TNF-α, as well as the immunosuppressive cytokine IL-10, were measured in macrophage supernatants after infection with distinct GBS isolates (**Figure 4**).

Three hours post-infection, IL-6 production varied significantly between isolates. The highest IL-6 concentrations were detected in macrophages infected with the colonizing serotype Ia isolate (9427) and two invasive serotype V isolates (104, 123), while macrophages infected with serotype III isolates (231, 9731) produced the lowest IL-6 levels at this early time point (**Figure 4A**). By 24 hours post-infection, IL-6 concentrations increased substantially in all conditions, with the highest levels observed following infection with serotype II (211, 7339) and serotype III (231, 9731) isolates, indicating a delayed but robust IL-6 response for these serotypes. TNF-α and IL-12 production also increased over time but showed distinct early-phase dynamics. While TNF-α concentrations were relatively uniform across isolates at 3 hours post-infection, IL-12 levels varied considerably, suggesting early isolate-specific modulation of IL-12 production (**Figures 4B, C**). At 24 hours post-infection, macrophages infected with serotype II and III isolates exhibited the highest concentrations of both IL-12 and TNF-α, indicating a late enhancement of the pro-inflammatory response for these isolates.

IL-10 production also showed a distinct temporal and serotype-dependent pattern. At 3 hours post-infection, the highest IL-10 concentrations were observed in macrophages infected with serotype Ia (9427, 229) and serotype Ib (203, 10276) isolates. By 24 hours post-infection, IL-10 production was highest after infection with serotype III isolates (231, 9731), the invasive serotype Ib isolate (203), and the colonizing serotype II isolate (7339) (**Figure 4D**), indicating a delayed induction of immunosuppressive responses for selected isolates.

To assess the relationship between IL-12 production and co-stimulatory signaling, cytokine data were further stratified by the expression of the co-stimulatory molecules CD80 and CD86, which were quantified in our previous study (Janzic et al., 2023). Macrophages with increased CD80 expression produced significantly higher levels of IL-12 at 24 hours PI, whereas no difference was observed at 3 hours post-infection (**Figure 4E**). However, no statistically significant association was found between IL-12 production and CD86 expression.

Cytokine responses were also examined in relation to the gestational age of the neonates from whom the invasive GBS isolates were obtained. Macrophages infected with isolates from preterm infants produced significantly higher levels of IL-10 at 3 hours post-infection compared with macrophages infected with isolates from term-born infants (**Figure 4F**). In contrast, IL-12 production was lowest following infection with isolates from moderate preterm infants and reached the highest levels at 24 hours post-infection with isolates from term-born infants (**Figure 4G**).

Together, these data demonstrate that GBS infection induces highly dynamic, isolate-specific macrophage cytokine responses, characterized by distinct temporal patterns of pro-inflammatory and immunosuppressive cytokine production. The magnitude and kinetics of these responses are strongly influenced by bacterial serotype, evolve over time, and are further shaped by co-stimulatory signaling capacity and neonatal gestational age, likely reflecting adaptation to different host environments.

### 3.5 Cytokine responses vary depending on the clinical presentation and specimen type, suggesting site-specific immune activation

**Figure 5:**
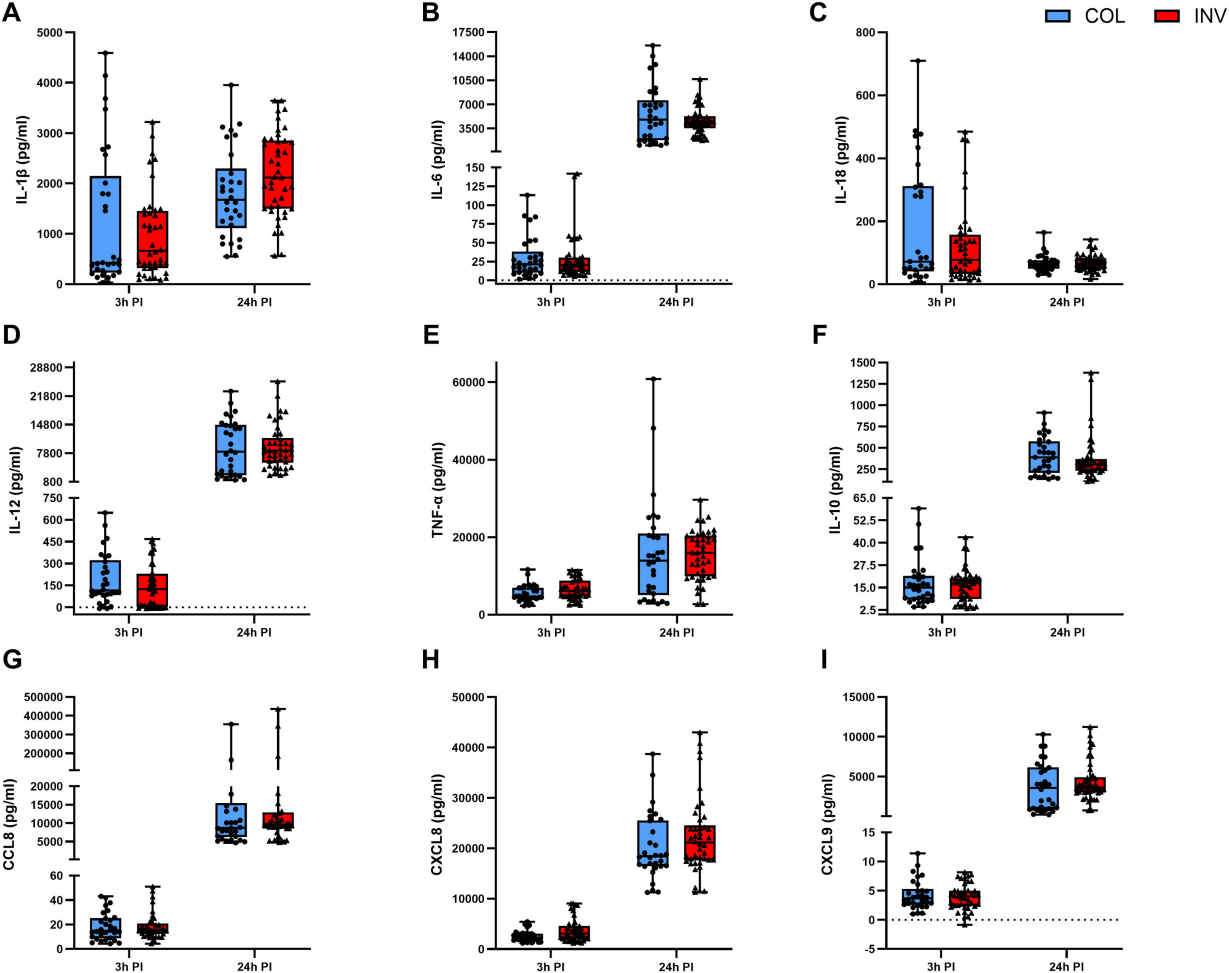
Production of cytokines and chemokines in macrophages stimulated with GBS isolates with different clinical presentations. The graphs show the mean values ± SEM of the inflammatory cytokines **(A)** IL-1β, **(B)** IL-6, **(C)** IL-18, **(D)** IL-12, **(E)** TNF-α; **(F)** the immunosuppressive cytokine IL-10 and the chemokines **(G)** CCL8 (MCP2), **(H)** CXCL8 (IL-8) and **(I)** CXCL9 (MIG), measured in the supernatants of infected macrophages in three independent biological replicates with two technical replicates each (n = 6). **p ≤ 0.01, determined by Kruskal-Wallis test and *post-hoc* Dunn’s multiple comparison test. PI, post-infection.

**Figure 6:**
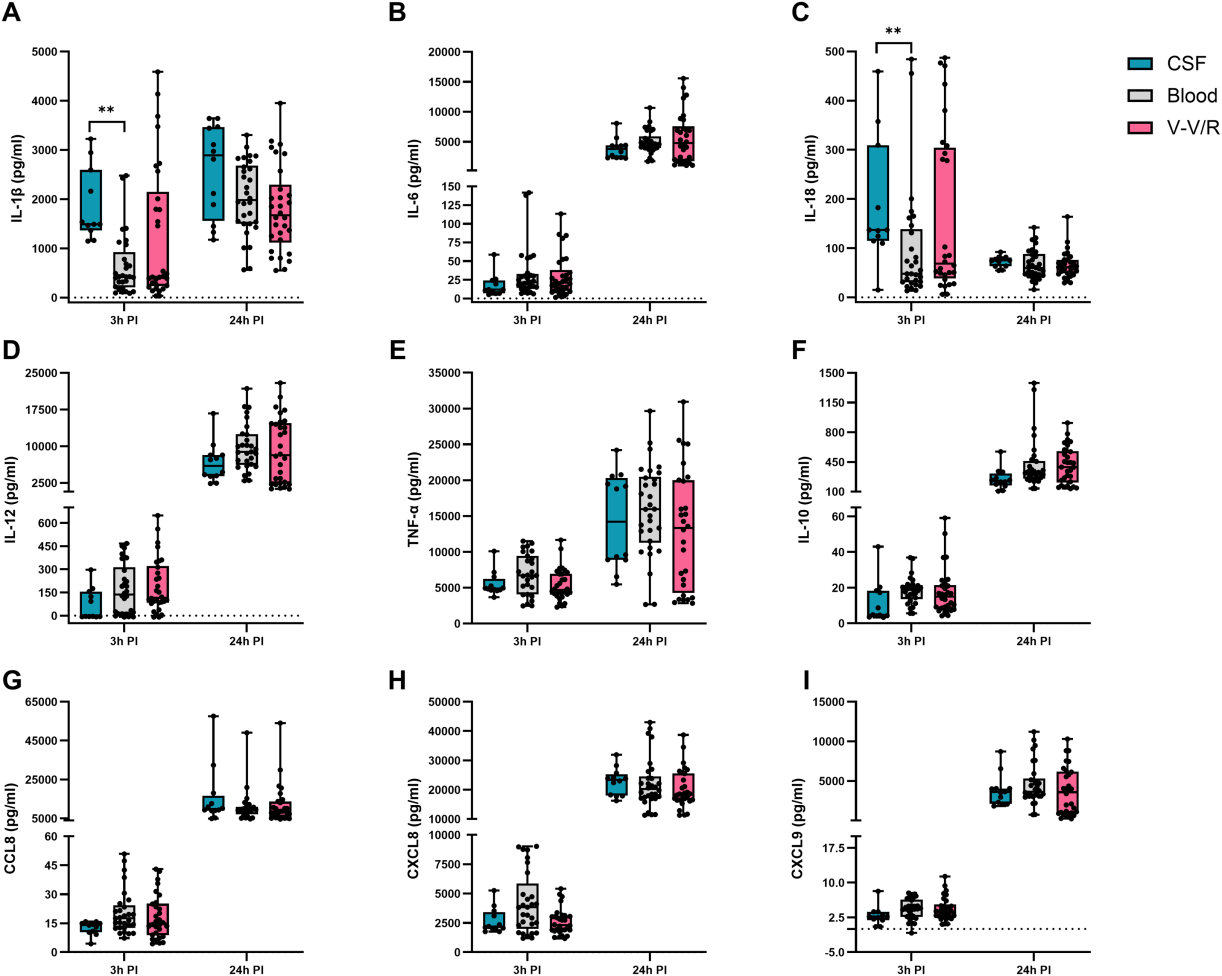
Production of cytokines and chemokines in macrophages stimulated with GBS isolates from different specimens. The graphs show the mean values ± SEM of the inflammatory cytokines **(A)** IL-1β, **(B)** IL-6, **(C)** IL-18, **(D)** IL-12, **(E)** TNF-α; **(F)** the immunosuppressive cytokine IL-10 and the chemokines **(G)** CCL8 (MCP2), **(H)** CXCL8 (IL-8) and **(I)** CXCL9 (MIG), measured in the supernatants of infected macrophages in three independent biological replicates with two technical replicates each (n = 6). Statistically significant differences were determined using the Kruskal-Wallis test and Dunn’s multiple comparison test and are summarized in **Supplementary Table S19–S27** for clarity. CSF, cerebrospinal fluid; V/V-R, vagina/vagina-rectum; PI, post-infection. **p ≤ 0.01.

To evaluate how the clinical context influences host immune responses, data were further stratified by clinical presentation of GBS isolates (colonizing (blue) vs. invasive (red)), (**Figure 5**) and specimen type (blood, cerebrospinal fluid (CSF) or vagina/vagina-rectum (V/V-R)), (**Figure 6**). Although no statistically significant differences were found between colonizing and invasive isolates at individual time points, clear trends were observed. For example, slightly higher levels of IL-1β and IL-18 were observed 3 hours post-infection with colonizing isolates (**Figures 5A, C**), while TNF-α concentrations were slightly increased in response to invasive isolates, but the differences decreased after 24 hours (**Figure 5E**).

In contrast, cytokine production differed significantly when data were stratified by specimen type, as summarized in **Supplementary Tables S19–S27**. Macrophages exposed to CSF isolates produced significantly higher levels of IL-1β and IL-18 compared to those infected with isolates from blood or V/V-R (**Figures 6A, C**), but after 24 hours this pattern was no longer significant, suggesting an early, transient inflammatory burst specific to CSF isolates. Conversely, isolates from blood elicited significantly higher early production of IL-6, TNF-α, and the chemokines CCL8, CXCL8 and CXCL9 compared to isolates from CSF or V/V-R (**Figures 6B, E, G–I**).

### 3.6 Integrated serotype-specific inflammatory and immunosuppressive response signatures revealed by radar plot analysis

**Figure 7:**
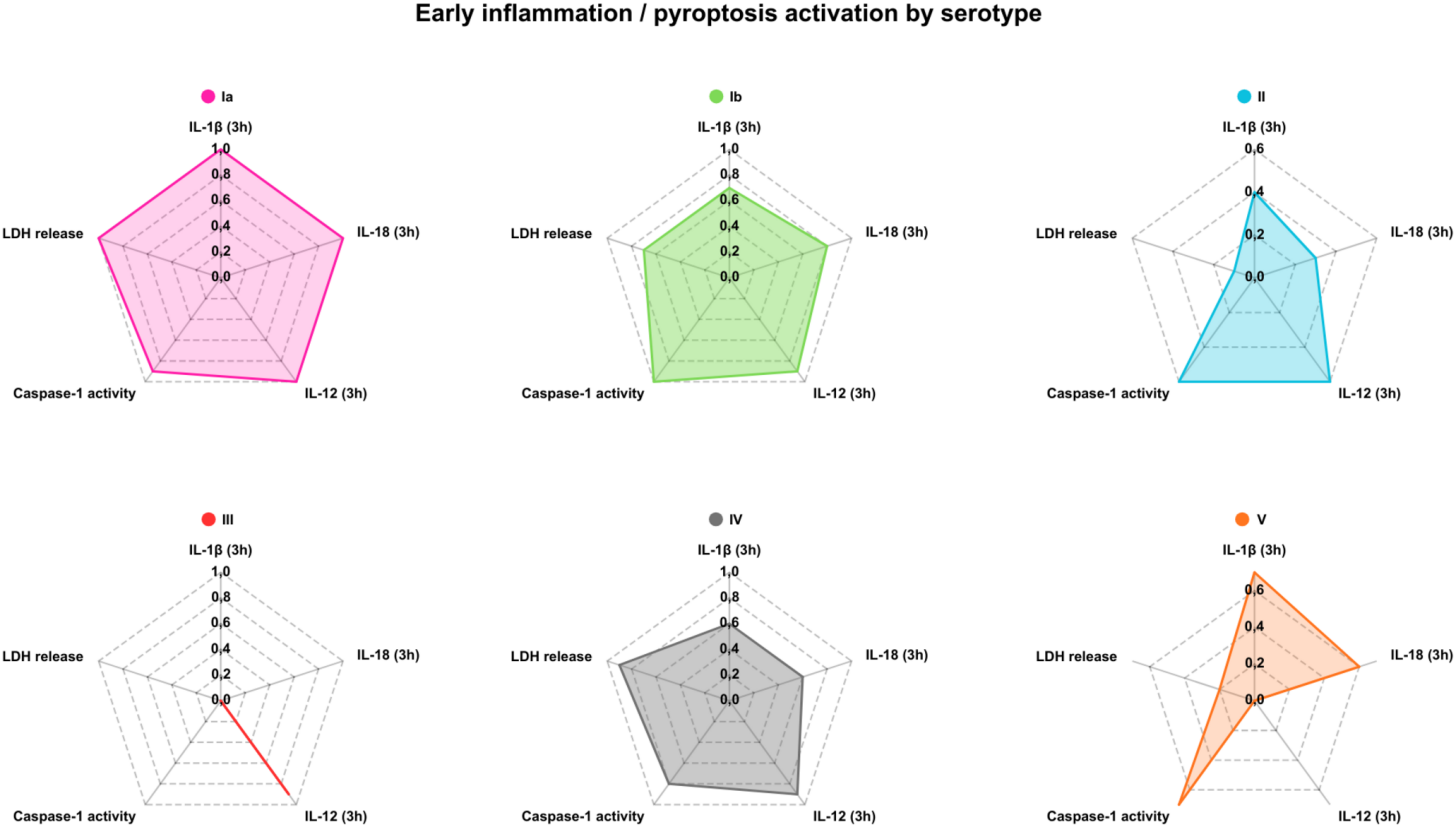
Serotype-specific early inflammasome and pyroptosis-associated responses in human macrophages infected with Group B *Streptococcus*. Radar plots summarize early (3 hours post-infection) macrophage responses to clinical GBS isolates, grouped by serotype (Ia, Ib, II-V). Each plot shows normalized values for IL-1β, IL-18, IL-12, caspase-1 activity, and macrophage lysis (LDH release), representing key indicators of inflammasome activation and pyroptotic cell death. Values are scaled to the maximum response observed across serotypes for each parameter, allowing direct visual comparison of serotype-specific immune activation profiles.

**Figure 8:**
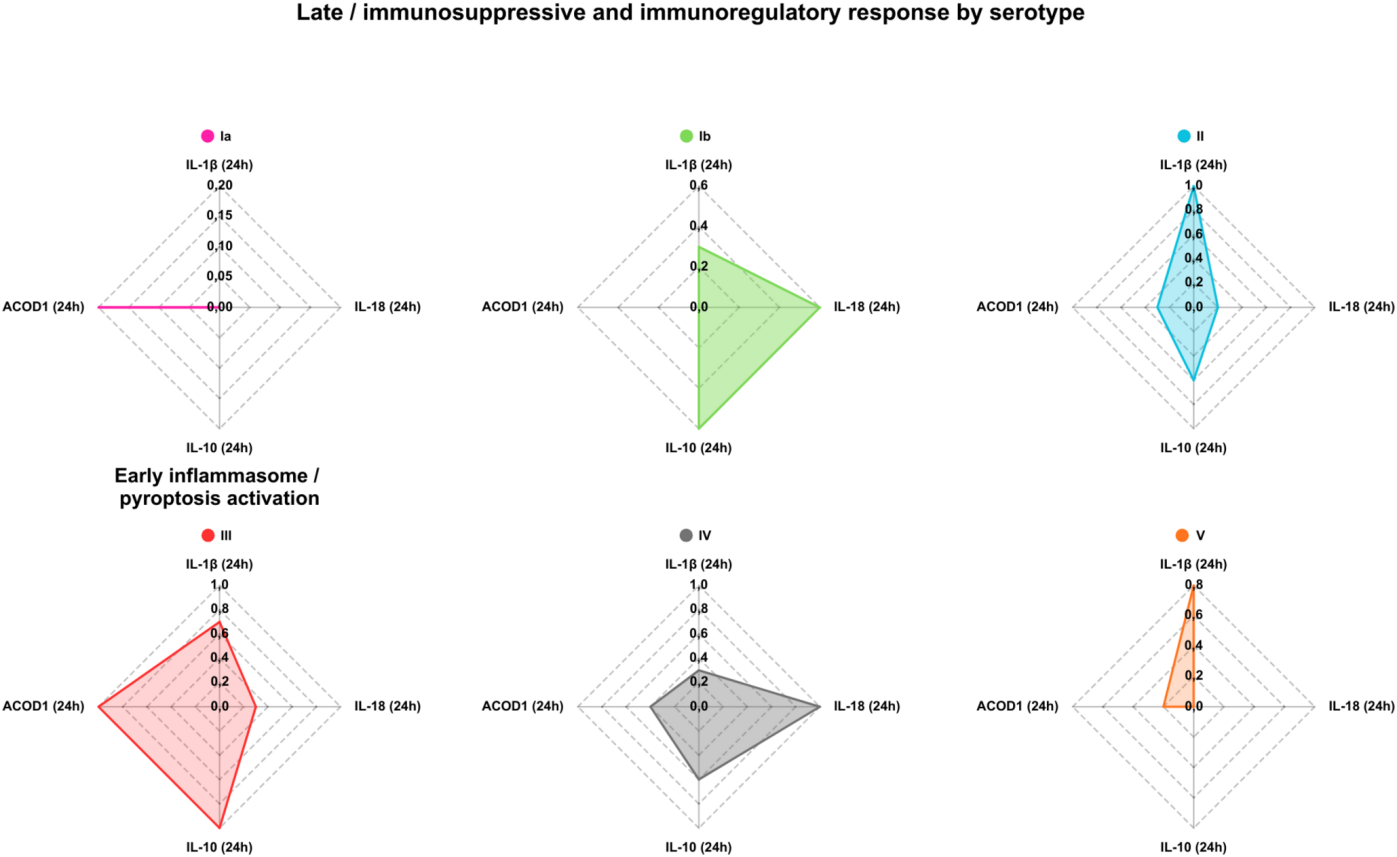
Serotype-specific late immunoregulatory responses in human macrophages infected with Group B *Streptococcus*. Radar plots summarize macrophage responses at 24 hours post-infection with clinical GBS isolates, grouped by serotype (Ia, Ib, II- V). Each plot shows normalized values for IL-1β, IL-18, IL-10, and *ACOD1* expression, reflecting the balance between residual inflammatory signaling and the induction of immunoregulatory and metabolic pathways at later stages of infection. Values are scaled to the maximum response observed across serotypes for each parameter, allowing direct comparison of serotype-specific late immune response profiles.

To provide an integrated overview of early inflammatory and late immunoregulatory macrophage responses induced by different GBS serotypes, key cytokines, cell death, and metabolic readouts were visualized using radar plots that summarize normalized responses across isolates within each serotype (**Figures 7**, **8**).

At 3 hours post-infection, serotype Ia isolates exhibited a coordinated early inflammatory signature characterized by high IL-1β and IL-18 production, elevated caspase-1 activity, pronounced LDH release, and strong IL-12 induction, consistent with rapid and robust early inflammasome engagement and pyroptosis. Serotype Ib isolates showed a similar but less pronounced profile. In contrast, serotype II isolates demonstrated partial early activation, with relatively high IL-12 and caspase-1 activity but reduced IL-18 production and LDH release, indicating limited early cell lysis. Serotype IV isolates induced intermediate responses across parameters, while serotype V isolates were heterogeneous, with some showing strong caspase-1 activity and IL-18 production but comparatively low LDH release. Notably, serotype III isolates displayed minimal early induction of IL-1β, IL-18, caspase-1 activity, and LDH release despite detectable IL-12 production, indicating a markedly attenuated early inflammatory and pyroptotic response (**Figure 7**).

At 24 hours post-infection, distinct late immunosuppressive and immunoregulatory response patterns emerged. Serotype III isolates were marked by strong induction of IL-10 and *ACOD1*, along with low IL-18 levels, indicating a shift toward immunoregulatory and metabolic programs. Serotype II isolates also showed elevated late IL-10 and IL-1β, but with weaker *ACOD1* induction, reflecting a mixed inflammatory–immunosuppressive phenotype. In contrast, serotype Ia isolates showed minimal late IL-10 and *ACOD1* induction, consistent with a predominantly early inflammatory response. Serotype Ib isolates displayed moderate late IL-10 and IL-18 production, while serotype IV isolates showed balanced but modest late responses. Serotype V isolates again demonstrated heterogeneity, with some isolates exhibiting elevated late IL-1β without strong induction of immunosuppressive markers (**Figure 8**).

### 3.7 Gene expression profiling reveals isolate-specific reciprocal regulation of *IL1B* and *ACOD1* and its association with *IL10* expression in GBS-infected macrophages

**Figure 9:**
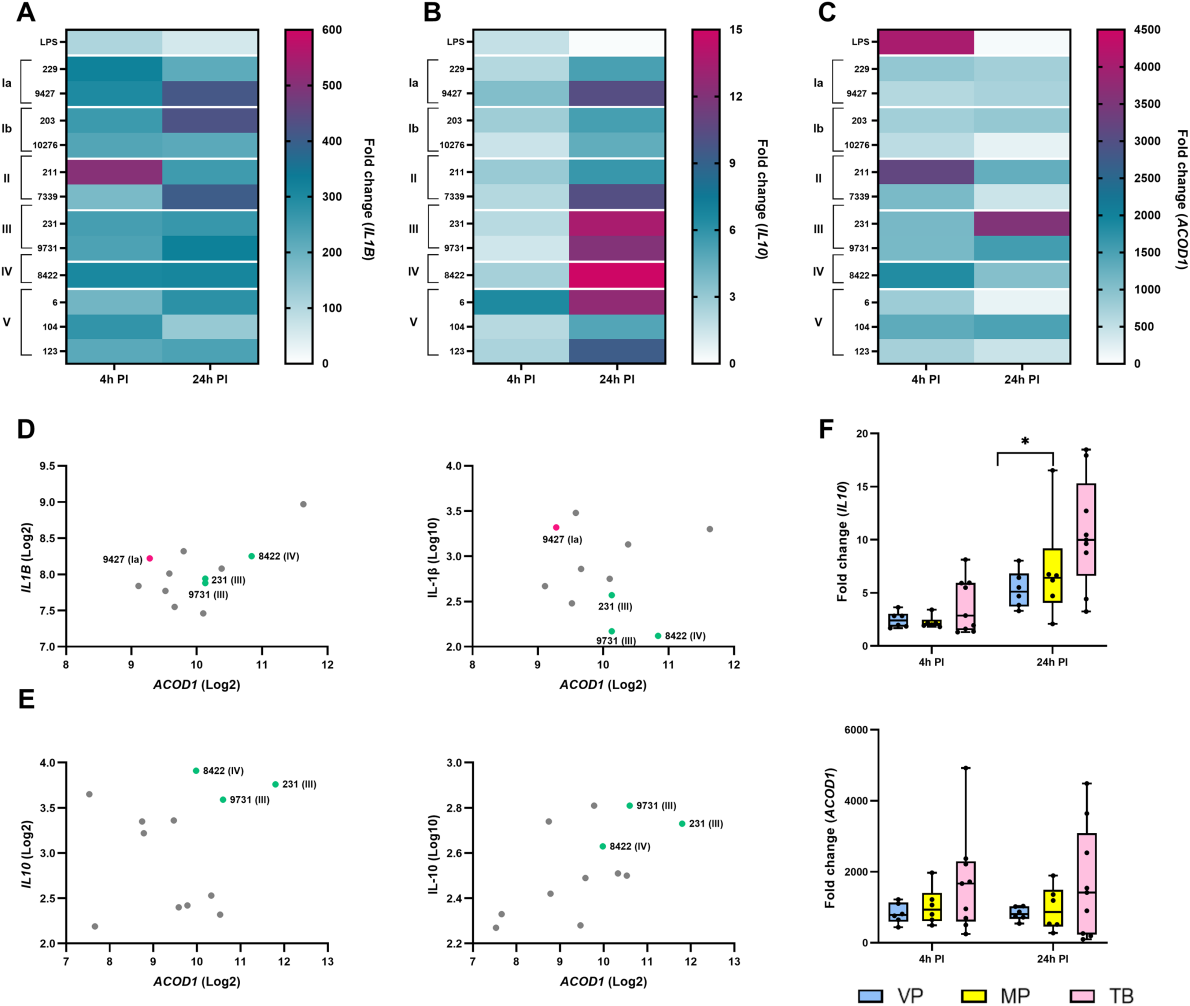
Reciprocal patterns of inflammatory and immunoregulatory gene expression and their association with neonatal gestational age. RNA was isolated from infected macrophages at 4 hours and 24 hours after infection with 12 different GBS isolates, and gene expression was determined by RT-qPCR. The heatmaps show the fold change in gene expression compared to unstimulated control macrophages for genes encoding: **(A)** the highly inflammatory cytokine IL-1β; **(B)** the immunosuppressive cytokine IL-10; and **(C)** the enzyme cis-aconitate decarboxylase, which is responsible for the formation of the antimicrobial and immunosuppressive itaconate. Pink/purple indicates higher expression; green indicates lower expression. All experiments were performed in three independent biological replicates, each with two technical replicates (n = 6). Statistically significant differences between macrophages stimulated with individual GBS isolates are summarized in **Supplementary Tables S28–S33** and were determined by one-way ANOVA and *post-hoc* Šidák’s multiple comparison test. **(D)** Reciprocal correlation plots show the correlation between *ACOD1* and IL-1β expression at the mRNA level (left) or protein level (right) at 4 hours post-infection. **(E)** Reciprocal correlation plots show the correlation between *ACOD1* and IL-10 expression at the mRNA level (left) or protein level (right) at 24 hours post-infection. **(F)** Expression of *IL10* (top) and *ACOD1* (bottom) according to the gestational age of the neonates (VP, severely/very preterm [28 to <32 weeks]; MP, moderately preterm [32 to <37 weeks]; TB, term born [over 37 weeks]). *p ≤ 0.05 and **p ≤ 0.01, determined by unpaired t-test or Mann-Whitney test. PI, post-infection.

To further characterize the transcriptional programs underlying GBS-induced macrophage responses, the expression of key inflammatory and immunoregulatory genes (*IL1B, IL10*, and *ACOD1*) was analyzed by RT-qPCR at 4 hours and 24 hours post-infection. Gene expression levels were normalized to unstimulated controls and are presented as fold changes (**Figure 9**). Statistically significant differences between macrophages stimulated with individual isolates are shown in **Supplementary Tables S28–S33** for clarity.

Expression of *IL1B* was strongly upregulated in macrophages infected with all GBS isolates compared with unstimulated controls, confirming robust induction of inflammatory gene transcription. The highest *IL1B* expression at 4 hours post-infection was observed following infection with an invasive serotype II isolate (211), reaching nearly 600-fold induction, followed by isolates of serotype Ia (**Figure 9A**). At 24 hours post-infection, *IL1B* expression remained markedly elevated, with the highest levels detected in macrophages infected with colonizing isolates of serotypes Ia (9427) and II (7339), as well as an invasive isolate of serotype Ib (203). These transcriptional patterns were consistent with the pronounced IL-1β protein production observed, especially for isolates of serotypes Ia and Ib.

In contrast, expression of the immunosuppressive *IL10* showed a delayed, isolate-specific pattern. While modest induction was observed at 4 hours post-infection, *IL10* expression increased substantially by 24 hours, particularly in macrophages infected with serotype III and IV isolates, both of which carry the hypervirulence-associated HvgA gene (**Figure 9B**). Notably, the strongest and most consistent *IL10* upregulation at both time points (approximately eightfold) was induced by a CSF-derived invasive serotype V isolate (isolate 6), indicating pronounced activation of immunoregulatory transcriptional programs by selected isolates.

Expression of *ACOD1*, which encodes the enzyme IRG1 involved in metabolic regulation and linked to NLRP3 inhibition via itaconate production, showed marked temporal and isolate-dependent variation (**Figure 9C**). At 4 hours post-infection, *ACOD1* expression was highest in macrophages infected with the invasive serotype II isolate (211), whereas at 24 hours post-infection *ACOD1* expression peaked following infection with serotype III isolates (231, 9731). In contrast, serotype Ia and Ib isolates, which induced strong *IL1B* expression and robust early inflammatory responses, elicited only weak *ACOD1* induction at both time points.

To directly examine the relationship between inflammatory and immunoregulatory programs, reciprocal correlation plots were generated to show how *ACOD1* mRNA expression relates to inflammatory and immunoregulatory readouts at both the transcriptional and protein levels (**Figures 9D, E**). In panel D, *ACOD1* expression (mRNA) was plotted against *IL1B* expression (mRNA; left) and against IL-1β concentrations measured in supernatants (protein; right), revealing a negative association between *ACOD1* and IL-1β responses. Isolates that strongly induced *IL1B* expression and IL-1β secretion (especially serotype Ia) were consistently associated with low *ACOD1* expression, whereas isolates with high *ACOD1* expression (serotype III) exhibited comparatively reduced *IL1B* expression and IL-1β production. In panel E, *ACOD1* expression (mRNA) was plotted against *IL10* expression (mRNA; left) and against IL-10 concentrations measured in supernatants (protein; right). In contrast to IL-1β, these plots demonstrated a positive association between *ACOD1* and IL-10, with higher ACOD1 expression corresponding to higher *IL10* mRNA levels and increased IL-10 secretion, most evident for isolates of serotype III. Together, the reciprocal plots indicate that *ACOD1* expression is associated with an immunoregulatory phenotype characterized by elevated IL-10 production, while being inversely related to IL-1β–driven inflammatory responses across GBS isolates. This reciprocal pattern, observed particularly for isolates of serotypes Ia, Ib, and III, is mechanistically consistent with itaconate-mediated inhibition of NLRP3, the downstream consequence of *ACOD1* (IRG1) induction, and the resulting restriction of IL-1β and IL-18 production.

Finally, gene expression data were examined in relation to the gestational age of the neonates. Although the differences did not reach statistical significance, macrophages infected with isolates from term-born infants consistently showed higher expression of *IL10* and *ACOD1* compared with those infected with isolates from preterm infants, which tended to induce lower expression of both genes (**Figure 9F**).

### 3.8 Isolate-specific induction of glycolytic gene expression reflects metabolic reprogramming in GBS-infected macrophages

**Figure 10:**
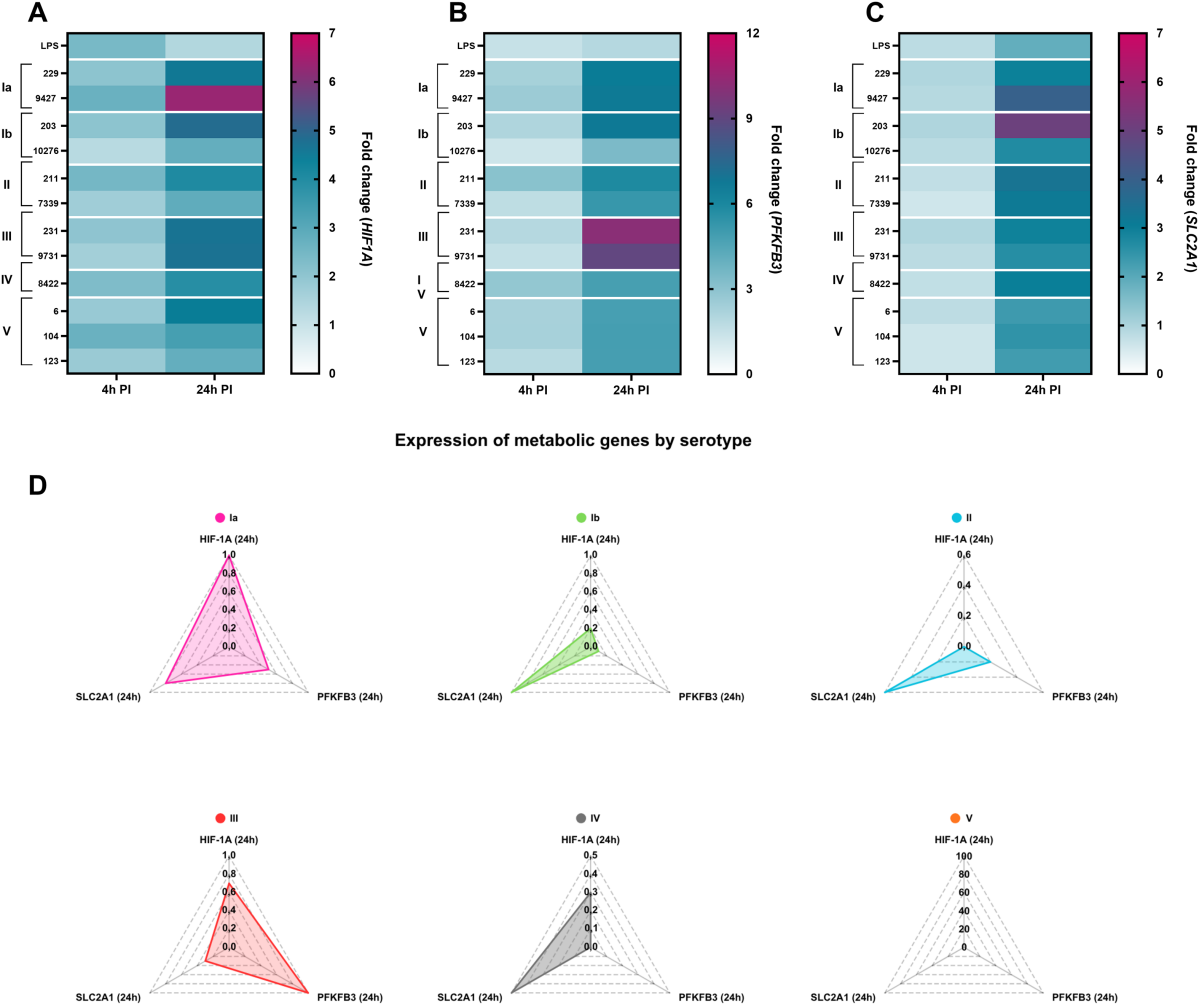
Distinct GBS isolates differentially modulate macrophage metabolic gene expression. RNA was isolated from infected macrophages 4 hours and 24 hours after infection, and metabolic gene expression was determined by RT-qPCR. The heatmaps show the fold change in gene expression compared to unstimulated control macrophages for genes encoding: **(A)** the transcription factor HIF-1α; **(B)** the enzyme PFKFB3; and **(C)** the glucose transporter GLUT1. All experiments were performed in three independent biological replicates, each with two technical replicates (n = 6). Statistically significant differences between macrophages stimulated with individual GBS isolates are summarized in **Supplementary Tables S34–S38** and were determined by one-way ANOVA with *post-hoc* Šidák’s multiple comparison test. **(D)** Radar plots summarize serotype-specific expression patterns of key metabolic genes in macrophages 24 hours after infection. Each plot represents a distinct GBS serotype (Ia, Ib, II, III, IV, and V) and summarizes the relative expression of *HIF1A, PFKFB3*, and *SLC2A1* measured by RT-qPCR. Gene expression values are normalized and scaled within each serotype to visualize the relative contributions of individual metabolic pathways. PI, post-infection.

To assess metabolic reprogramming in macrophages following infection with different GBS isolates, the expression of genes involved in glucose metabolism and its regulation (*HIF1A, PFKFB3*, and *SLC2A1 (GLUT1)*) was quantified by RT-qPCR at 4 hours and 24 hours post-infection and normalized to unstimulated controls (**Figure 10**).

At 4 hours post-infection, expression of all three metabolic genes remained low and showed minimal variation across isolates, indicating limited early transcriptional reprogramming. In contrast, at 24 hours post-infection a pronounced upregulation of glycolytic genes was observed, with marked isolate- and serotype-specific differences (**Figure 10**; **Supplementary Tables S34–S38**).

Expression of *HIF1A* increased substantially at 24 hours post-infection with serotype Ia (9427, 229) and serotype Ib (203) isolates, reaching approximately sevenfold induction compared with unstimulated macrophages (**Figure 10A**). Strong *HIF1A* induction was also observed following infection with serotype III isolates, whereas stimulation with LPS resulted in only minimal changes in *HIF1A* expression. The isolates inducing the highest *HIF1A* expression corresponded to those previously associated with elevated inflammatory and cell death markers, suggesting a link between inflammatory stress and activation of hypoxia- and glycolysis-related transcriptional programs.

Similarly, *PFKFB3*, a key regulator of glycolytic flux, was robustly induced at 24 hours post-infection, with the highest expression observed in macrophages infected with serotype III isolates (231, 9731), consistent with the enhanced glycolytic activity previously observed in Seahorse-based metabolic flux analyses in our earlier study (Janzic et al., 2023) (**Figure 10B**). Serotype Ia and Ib isolates also induced significant *PFKFB3* upregulation, with increases of approximately 6 to 8-fold.

Expression of the glucose transporter gene *SLC2A1 (GLUT1)* was also significantly increased at 24 hours post-infection, particularly in macrophages infected with serotype Ia and Ib isolates (**Figure 10C**). The induction of *SLC2A1* closely mirrored the expression profiles of *HIF1A* and *PFKFB3*, indicating coordinated activation of glucose uptake and glycolytic pathways in response to selected GBS isolates.

To provide an overview of metabolic gene expression patterns by serotype, radar plots were generated to summarize normalized *HIF1A, PFKFB3*, and *SLC2A1* expression across isolates of the same serotype (**Figure 10D**). These plots revealed distinct metabolic signatures. Serotype Ia showed a coordinated metabolic response with concurrent induction of *HIF1A, PFKFB3*, and *SLC2A1*, consistent with strong glycolytic reprogramming. Serotype III isolates exhibited a dominant *PFKFB3*-driven signature, with robust induction of glycolytic flux regulators and increased *HIF1A* expression. In contrast, serotypes II and IV showed more limited or unbalanced metabolic activation, with modest induction of individual genes but no strong coordinated glycolytic profile. Serotype V isolates showed comparatively weak induction of all three metabolic genes, indicating minimal metabolic reprogramming at the transcriptional level.

Together, these results show that GBS-induced metabolic reprogramming of macrophages is highly dependent on isolate and serotype, arises mainly at later stages of infection, and involves differential activation of glycolysis-associated gene networks. These distinct metabolic signatures indicate that individual GBS strains engage host metabolic pathways to varying degrees, potentially contributing to divergent inflammatory and immunoregulatory outcomes.

### 3.9 Heterogeneous caspase-mediated cell death responses to GBS infection

**Figure 11:**
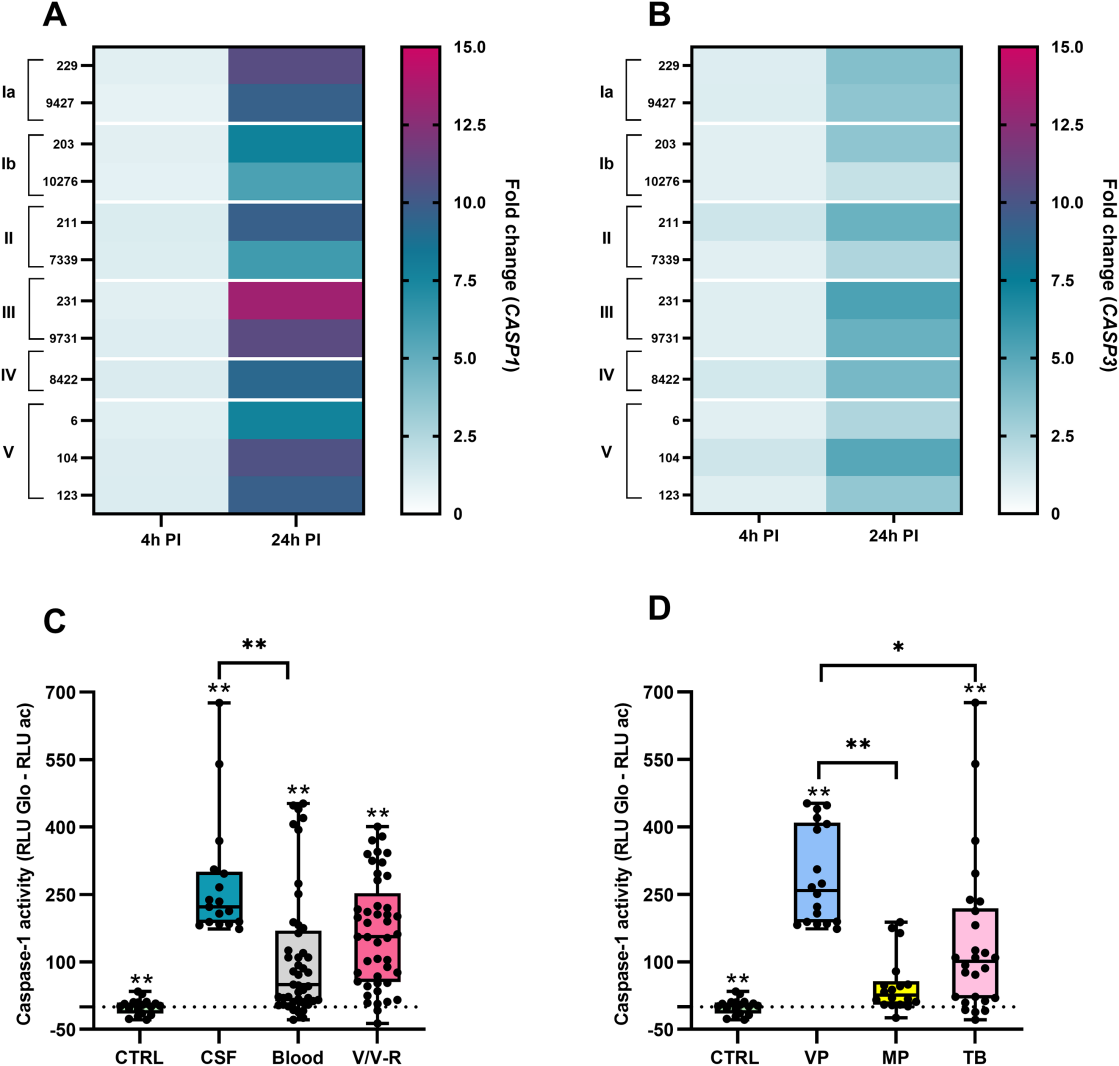
Differential expression of caspase-1 and caspase-3, and caspase-1 activity by specimen type and gestational age. RNA was isolated 4 hours and 24 hours after infection, and gene expression was determined by RT-qPCR. The heatmaps show the fold change in gene expression compared to unstimulated control macrophages for genes encoding: **(A)** caspase-1; **(B)** caspase-3. All experiments were performed in three independent biological replicates, each with two technical replicates (n = 6). Statistically significant differences between macrophages stimulated with individual GBS isolates are summarized in **Supplementary Tables S39–S43** and were determined by one-way ANOVA with *post-hoc* Šidák’s multiple comparison test. Box plots show caspase-1 activity according to **(C)** specimen type (CSF, cerebrospinal fluid; V/V-R, vagina/vagina-rectum) and **(D)** gestational age of the neonates (VP, severely/very preterm [28 to <32 weeks], MP, moderately preterm [32 to <37 weeks], TB – term born [over 37 weeks]). *p ≤ 0.05 and **p ≤ 0.01, determined by Kruskal-Wallis test and Dunn’s multiple comparison test. PI, post-infection; RLU, relative light units.

To characterize cell death–associated responses induced by GBS, the expression of *CASP1* and *CASP3*, which encode caspase-1 and caspase-3, respectively, was analyzed by RT-qPCR at 4 hours and 24 hours post-infection (**Figure 11**).

Expression of *CASP1*, which encodes a key enzyme involved in inflammatory cell death (pyroptosis), was low at 4 hours post-infection but increased markedly by 24 hours in macrophages infected with different GBS isolates (**Figure 11A**). Notably, elevated *CASP1* expression at 24 hours was also observed in macrophages that exhibited low caspase-1 activity and minimal cell lysis, indicating delayed transcriptional upregulation independent of early enzymatic activity. Expression of *CASP3*, which encodes caspase-3 involved in programmed cell death (apoptosis), also increased over time, although the magnitude of induction was lower than that observed for *CASP1* (**Figure 11B**). At 24 hours post-infection, the highest *CASP3* expression was detected in macrophages infected with serotype III isolates. Across all conditions, *CASP1* and *CASP3* expression levels showed a positive association, consistent with caspase activity measurements indicating concurrent engagement of inflammatory and apoptotic pathways in response to selected GBS isolates.

Caspase-1 activity was also assessed using a bioluminescence-based assay and stratified by specimen type and gestational age of the neonates (**Figures 11C, D**). All GBS isolates induced significantly higher caspase-1 activity compared with unstimulated macrophages, regardless of specimen type. Among these, CSF-derived isolates elicited the highest caspase-1 activity, followed by vaginal/rectal and blood-derived isolates (**Figure 11C**). Stratification by gestational age showed significantly higher caspase-1 activity in macrophages infected with isolates from very preterm (VP) neonates compared with those from moderately preterm (MP) or term-born (TB) infants (**Figure 11D**). These findings indicate that isolates associated with preterm birth are linked to increased caspase-1–mediated inflammatory signaling.

**Figure 12:**
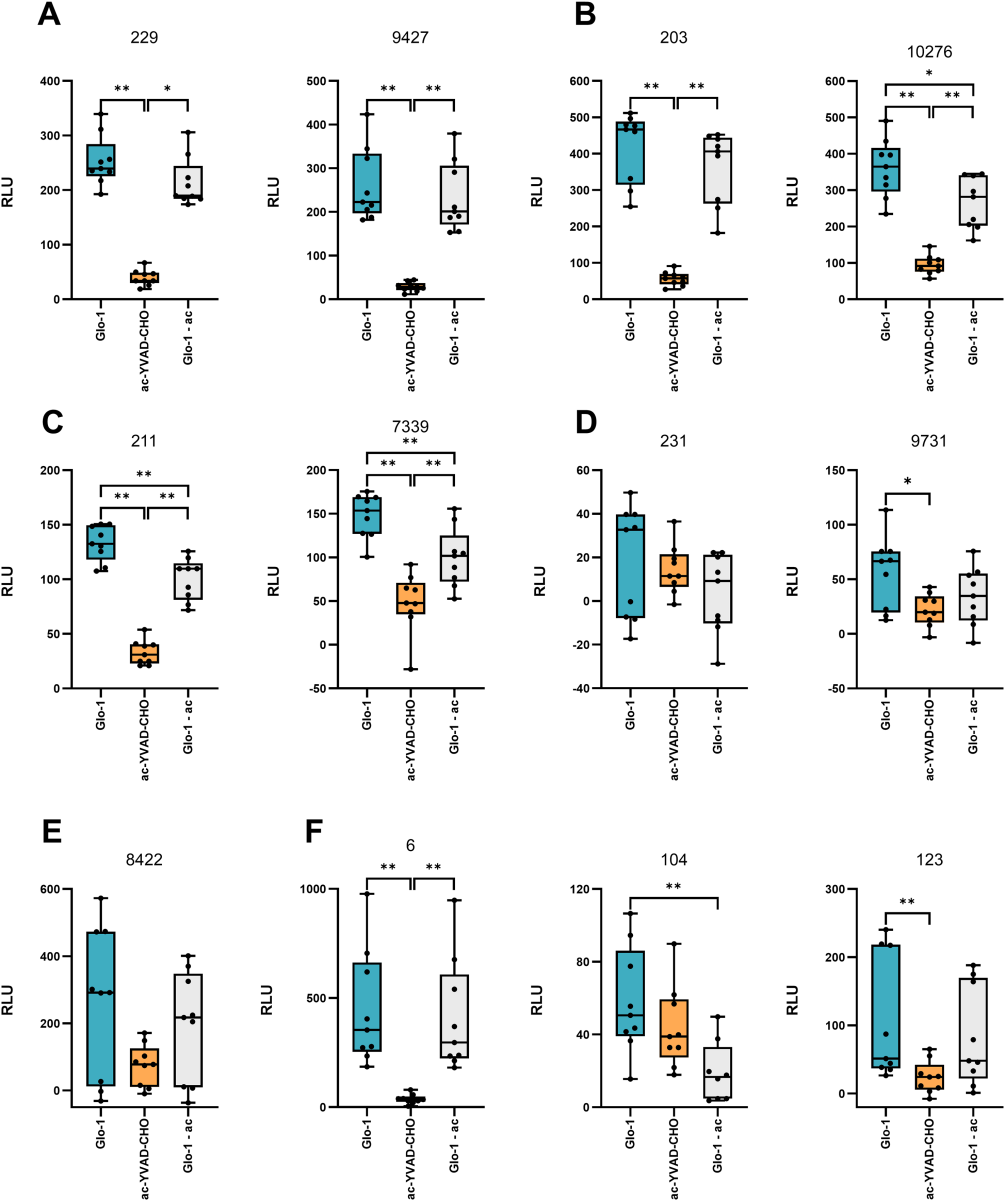
Caspase activity in macrophages infected with individual GBS isolates. The boxplots show the total caspase activity (Glo-1, shown in blue), the caspase activity after addition of a selective caspase-1 inhibitor, ac-YVAD-CHO (shown in orange) and the specific caspase-1 activity determined by subtracting the activity after addition of the inhibitor from the total caspase activity (Glo-1 - ac, shown in gray) for macrophages infected with isolates of serotypes **(A)** Ia, **(B)** Ib, **(C)** II, **(D)** III, **(E)** IV, and **(F)** V, respectively. All experiments were performed in three biological replicates, each with three technical replicates (n = 9). Statistically significant differences were determined using one-way ANOVA and Šidák *post-hoc* multiple comparison test or Kruskal-Wallis test with Dunn’s multiple comparison test. *p ≤ 0.05 and **p ≤ 0.01. RLU, relative light units.

**Figure 12** shows total caspase activity, residual caspase activity measured after the addition of the caspase-1–selective inhibitor ac-YVAD-CHO, and the calculated specific caspase-1 activity for each individual GBS isolate.

## 4 Discussion

Invasive Group B *Streptococcus* (GBS) infections remain a major cause of neonatal morbidity and mortality worldwide and pose a serious threat not only to newborns but also to the elderly and immunocompromised individuals (Landwehr-Kenzel and Henneke, 2014). The limitations of current preventive strategies and the increasing emergence of antibiotic resistance highlight the need for a deeper understanding of isolate-specific host–pathogen interactions that drive disease severity and clinical outcomes. Macrophages are central sentinels of innate immunity during GBS infection; however, how genetically and clinically distinct GBS isolates differentially modulate macrophage inflammatory, immunoregulatory, and metabolic responses remains incompletely understood.

In this study, we build on our previous work characterizing phagocytic uptake, macrophage lysis, and phenotypic and metabolic changes induced by fully genotyped clinical GBS isolates (Janzic et al., 2023) by providing a comprehensive, time-resolved analysis of cytokine production, inflammasome-associated signaling, metabolic reprogramming, and caspase-mediated cell death pathways. By integrating multiple functional readouts across early and late time points, our results show that macrophage responses to GBS are highly isolate- and serotype-specific and follow distinct temporal patterns.

A key strength of this study is the explicit incorporation of temporal dynamics. While most previous studies assessed macrophage cytokine responses at a single time point (typically 18–24 hours post-infection) (Flaherty et al., 2021, Flaherty et al., 2019), we quantified cytokine secretion at both 3 hours and 24 hours post-infection and analyzed gene expression at 4 hours and 24 hours. This approach revealed marked temporal heterogeneity that would have been missed in single–time-point analyses. For example, inflammasome-associated cytokines IL-1β and IL-18 peaked rapidly in response to selected isolates and then declined, whereas cytokines such as IL-6 and IL-10 accumulated over time. These findings caution against inferring macrophage behavior from single isolates or single time points and emphasize that both early activation and later adaptation phases are critical for understanding GBS–macrophage interactions.

Our data strongly supports the hypothesis that macrophage responses to GBS are isolate-specific and reflect distinct bacterial virulence strategies. Serotype Ia and Ib isolates consistently induced robust early inflammatory responses, characterized by rapid IL-1β and IL-18 secretion, high caspase-1 activity, and extensive macrophage lysis – a hallmark of NLRP3 inflammasome-associated pyroptotic cell death (Booty and Bryant, 2022; Yu et al., 2021). These early responses were most prominent within the first 3 hours post-infection, suggesting rapid engagement and activation of the NLRP3 inflammasome and pyroptosis. Although pyroptosis leads to cell lysis and death, it is generally considered a host-protective mechanism that limits intracellular pathogen survival through inflammatory cell death, inflammation, and immune activation (Brokatzky and Mostowy, 2022). Animal studies in mouse models have demonstrated that caspase-1-dependent IL-1β production and caspase-1 activity are essential for optimal clearance of GBS, and deficiency in these pathways results in increased susceptibility to invasive disease (Costa et al., 2012; Gupta et al., 2014). Although the cytokine profiles and caspase-1 activity observed in this study strongly suggest inflammasome activation, the specific involvement of the NLRP3 inflammasome was not directly tested and should therefore be confirmed in future studies. Mechanistically, the potent early inflammatory response induced by serotype Ia and Ib isolates is likely linked to β-hemolysin/cytolysin activity, a well-established trigger of NLRP3 inflammasome activation. β-hemolysin induces phagolysosomal damage and cytosolic leakage of bacterial components such as bacterial RNA, leading to caspase-1 activation and IL-1β/IL-18 maturation (Gupta et al., 2014; Korir et al., 2017). Gupta et al. (2014) demonstrated that GBS-induced IL-1β production in macrophages is strictly dependent on the presence of β-hemolysin and NLRP3, as hemolysin-deficient GBS mutants do not induce IL-1β, and NLRP3-deficient cells also fail to activate caspase-1 to produce IL-1β (Gupta et al., 2014). Consistent with this mechanism, we observed that isolates eliciting the highest inflammasome-associated cytokine responses (serotype Ia and Ib) also displayed pronounced hemolytic activity on blood agar plates. However, although β-hemolysin/cytolysin has been identified as an important virulence factor involved in NLRP3 inflammasome activation in GBS infections, its expression or activity was not directly quantified in the present study. Therefore, the potential contribution of this toxin to the observed inflammasome responses should be interpreted cautiously. Notably, this early inflammatory burst was transient, as IL-1β and IL-18 levels stabilized or declined by 24 hours post-infection, likely reflecting the loss of pyroptotic macrophages and subsequent resolution or reprogramming of the response.

In contrast, serotype II and especially hypervirulent serotype III (ST-17 lineage) isolates elicited minimal early inflammasome-associated responses. At 3 hours post-infection, macrophages infected with these isolates produced little IL-1β or IL-18, exhibited low caspase-1 activity, and showed minimal lysis. This attenuated early response was not due to impaired bacterial uptake, as serotype III isolates displayed efficient phagocytosis in our previous study (Janzic et al., 2023). Instead, these findings suggest that hypervirulent strains actively suppress or delay inflammasome activation, allowing infected macrophages to remain viable during early infection. Such viable macrophages may serve as “Trojan horses,” facilitating bacterial dissemination while evading extracellular immune defenses.

This immune-evasive phenotype was further supported by the marked induction of immunoregulatory mediators, particularly *IL10* and *ACOD1* (IRG1), in response to serotype III isolates. *ACOD1* encodes cis-aconitate decarboxylase, which produces itaconate – a metabolite with both antimicrobial and anti-inflammatory properties. Itaconate exerts a bacteriostatic effect by inhibiting the bacterial glyoxylate cycle and thus bacterial growth, while also suppressing inflammation by inhibiting succinate dehydrogenase (SDH). This inhibition leads to succinate accumulation, reduced ROS production, stabilization of HIF-1α, and suppressed NLRP3 inflammasome activity, thereby restricting IL-1β and IL-18 production (Wu et al., 2022b). Consistent with this, previous studies reported that ST-17 strains can inhibit ROS formation and inflammation in macrophages (Flaherty et al., 2019; Korir et al., 2018), aligning with our finding that ST-17 induced the highest *ACOD1* expression and a dampened inflammasome response. Our reciprocal correlation analyses demonstrated a clear inverse relationship between *ACOD1* expression and IL-1β responses, along with a positive association between *ACOD1* and IL-10 at both the transcriptional and protein levels. These reciprocal patterns suggest that *ACOD1*-associated immunometabolic reprogramming contributes to dampening inflammatory responses, particularly in serotype III infections.

The radar plot analyses provided an integrated visualization of these divergent response programs. Early radar plots showed that serotype Ia and Ib isolates induced a coordinated inflammatory signature encompassing IL-1β, IL-18, caspase-1 activity, LDH release, and IL-12, consistent with rapid inflammasome engagement and pyroptosis. In contrast, serotype III isolates showed minimal early inflammatory activation despite detectable IL-12 production, highlighting selective engagement of innate signaling pathways. Late radar plots further illustrated that serotype III isolates shifted toward a dominant immunoregulatory profile characterized by high *IL10* and *ACOD1* expression, whereas serotype Ia isolates showed little late immunosuppressive induction. These integrated profiles underscore that GBS serotypes differ not only in the magnitude but also in the temporal structure of macrophage responses.

Beyond inflammasome-associated pathways, we observed substantial serotype-specific differences in broader cytokine networks. IL-12 and TNF-α production increased over time, particularly in response to serotype II and III isolates, suggesting activation of pattern-recognition receptor pathways independent of inflammasome signaling. Stratification by CD80/CD86 expression, quantified in our previous study (Janzic et al., 2023), revealed that increased CD80 expression was associated with higher IL-12 production at later time points, consistent with enhanced capacity for T-cell activation and adaptive immune engagement, as CD80 and CD86, together with IL-12, provide the co-stimulatory and cytokine cues that drive downstream immune responses by promoting T-cell activation (Landwehr-Kenzel and Henneke, 2014).

Our analysis also uncovered pronounced isolate-specific metabolic reprogramming of macrophages. Glycolysis-associated genes *HIF1A, PFKFB3*, and *SLC2A1* were strongly upregulated at 24 hours post-infection in an isolate- and serotype-dependent manner. Serotype Ia and Ib isolates induced coordinated upregulation of all three genes, indicating a shift toward accelerated Warburg-like metabolism. This metabolic reprogramming, characterized by increased glucose uptake and glycolysis, is a hallmark of classically activated (M1) macrophages and supports the rapid generation of ATP and biosynthetic precursors essential for an effective acute inflammatory response (Kelly and O'Neill, 2015; Amo-Aparicio et al., 2024). Notably, elevated levels of IL-1β modulate and upregulate the expression of *HIF1A* and *PFKFB3*, increasing glycolytic activity. This creates a forward loop in which IL-1β drives metabolic reprogramming toward increased glycolytic flux and further enhances the pro-inflammatory phenotype of activated macrophages (Jia et al., 2025; Finucane et al., 2019). Interestingly, serotype III isolates also induced strong glycolytic gene expression (particularly *PFKFB3*) despite weak inflammasome activation. This suggests that glycolytic reprogramming in response to ST-17 strains may be driven by NLRP3-independent pathways (e.g., TLR–mTOR/HIF-1α signaling), allowing macrophages to remain metabolically active while suppressing inflammatory cell death (Qiu et al., 2023).

Caspase gene expression analyses further highlighted the complexity of cell death regulation during GBS infection. *CASP1* and *CASP3* expression increased over time, with serotype III isolates inducing the highest *CASP3* levels at 24 hours. The positive association between *CASP1* and *CASP3* expression, together with caspase activity measurements, suggests that inflammatory and apoptotic pathways may be engaged concurrently in response to selected isolates. Excessive caspase-3 activity has been shown to suppress NF-κB signaling and inflammatory cytokine production, providing another potential immune evasion mechanism employed by hypervirulent strains (Wu et al., 2022a).

Stratification of responses by gestational age of the neonates and specimen type revealed clinically relevant patterns. Isolates from preterm infants tended to induce stronger early inflammasome-associated responses, with increased caspase-1 activity, excessive pyroptosis, and resulting inflammation, whereas isolates from term infants more frequently exhibited immunoregulatory profiles characterized by IL-10 and *ACOD1* induction. These immunomodulatory strategies may allow such strains to cause insidious infections, such as meningitis and sepsis, in otherwise healthy term infants by evading early immune defenses. Furthermore, our data suggest that the anatomical source of the isolate (blood, CSF, or vagina/vaginal rectum) may also influence macrophage responses, as invasive isolates from infected neonates (blood/CSF) often showed either extremely pro-inflammatory or profoundly evasive phenotypes. Although these findings are based on an *in vitro* model, they suggest that strain-specific innate immune activation may contribute to pathological inflammation and therefore disease severity and clinical presentation, including the risk of preterm birth, sepsis, or meningitis.

We are aware of the limitations of our study. First, all experiments were conducted in an *in vitro* macrophage infection model using THP-1–derived macrophages. Although this model is well established and reproducible, it does not fully represent the complexity of primary human macrophages or the *in vivo* immune environment, including interactions with other immune cells, tissue-specific cues, and systemic signals. Using this standardized model minimized donor-to-donor variability and ensured that observed differences in immune responses primarily reflected the heterogeneity of the GBS isolates rather than variability between primary cell donors. Future studies should validate these findings in primary human macrophages to further investigate strain-specific immune responses in a more physiologically relevant model. Second, although multiple readouts associated with inflammasome-related signaling were assessed, such as IL-1β and IL-18 production, caspase-1 activity, macrophage lysis, and transcriptional changes, direct genetic or pharmacological inhibitions of specific inflammasome components (e.g., NLRP3 or gasdermin D) were not performed. Therefore, while the observed patterns are consistent with NLRP3 inflammasome involvement, definitive causal attribution cannot be made and will require targeted inhibition or genetic approaches in our future studies. Moreover, as gene expression analyses were restricted to selected inflammatory, immunoregulatory, metabolic, and cell death–associated genes, broader transcriptomic or proteomic profiling could identify additional pathways contributing to isolate-specific macrophage reprogramming. Although a diverse panel of well-characterized clinical GBS isolates was analyzed, the number of isolates per serotype and clinical subgroup was limited, which may constrain the generalizability of serotype- or gestational age–associated trends. Larger and more diverse isolate collections will be required to validate these findings. Finally, while radar plots enabled integrative visualization of multidimensional immune responses, they represent normalized summaries of selected parameters and may obscure isolate-level variability; thus, they should be interpreted as complementary to detailed quantitative analyses. Despite these limitations, the use of multiple clinical isolates, temporal resolution, diverse functional readouts, and integrative analyses provides a robust framework for understanding heterogeneity in macrophage responses to GBS and supports future mechanistic and translational investigations.

In summary, our study shows that GBS isolates use at least two distinct host–immune interaction strategies: either provoking strong immune activation or evading early immune recognition. Serotype Ia and Ib isolates trigger a hyperinflammatory response marked by rapid inflammasome activation, inflammatory cell death, and excessive local inflammation. Although such responses can alert the immune system, they may also benefit the pathogen by overwhelming the immature neonatal immune response and causing collateral tissue damage that impairs effective clearance. In contrast, serotype II and especially serotype III isolates largely avoid early inflammasome activation and instead induce immunosuppressive mediators such as IL-10 and itaconate. These isolates also elicited the highest expression of *CASP3*, which encodes caspase-3; excessive activation of caspase-3 can suppress NF-κB signaling and inflammatory cytokine production, providing an additional immune-evasion mechanism (Wu et al., 2022a). By comparing multiple clinical GBS isolates under identical conditions, we demonstrate substantial heterogeneity in macrophage responses, with distinct isolate-specific activation “fingerprints.” This variability indicates that disease outcome depends not only on host immune status but also on the infecting bacterial strain, helping to explain the broad clinical spectrum from fulminant sepsis to late-onset disease. Using two time points also allowed us to distinguish between hyperinflammatory and immune-evasive isolates that might be missed in single-time-point analyses. Together, these findings suggest that characterizing the infecting GBS isolate could inform clinical risk assessment and support targeted interventions, such as anticipating immune evasion and invasive spread in ST-17 infections or excessive inflammation in highly hemolytic serotype Ia infections.

## 5 Conclusions

In conclusion, this study provides a comprehensive, temporally resolved analysis of isolate-specific macrophage responses to clinically relevant Group B *Streptococcus* strains and shows that GBS pathogenicity is shaped by dynamic interactions between strain-specific virulence traits and host innate immune programs. By integrating inflammatory, immunoregulatory, metabolic, and cell death–associated responses across genetically diverse isolates, we establish a unified framework for understanding how distinct immune trajectories develop over time. Importantly, our findings link early hyperinflammatory responses and delayed immune-evasive programs to isolates associated with preterm birth and invasive neonatal disease, offering mechanistic insight into the heterogeneity of neonatal sepsis outcomes. These results highlight the importance of incorporating temporal resolution and strain heterogeneity into host–pathogen studies and support a precision-based approach to GBS infection, in which stratifying isolates by inflammatory potential may inform targeted preventive and therapeutic strategies for vulnerable populations.

## Data Availability

The original contributions presented in the study are included in the article/**Supplementary Material**. Further inquiries can be directed to the corresponding author.
