# Supplementary material for: Not all Group B Streptococci are alike: macrophage responses reveal inflammatory and immune-evasive strains
Source: Front Cell Infect Microbiol. 2026 Apr 16;16:1819218. doi: 10.3389/fcimb.2026.1819218 (PMC13128655; doi:10.3389/fcimb.2026.1819218)
Supplement: Supplementary file 1 [file Table1.docx]

Supplementary Material

# Supplementary Data

Supplementary Tables S1-S43 with extended statistical information

**Table S1: Statistically significant differences in CXCL9 chemokine production 3 hours after macrophage infection with individual GBS isolates,** determined by one-way ANOVA with *post-hoc* Šidák test or by Kruskal-Wallis test with Dunn's test.

| **GBS isolates** | **Šidák/Dunn's test for multiple comparisons   (p-value)** |  | **GBS izolati** | **Šidák/Dunnetov test večkratne primerjave  (p-vrednost)** |
| --- | --- | --- | --- | --- |
| 9427 (ST-144) vs. 203 (ST-8) | 0.0204 |  | 203 (ST-8) vs. 6 (ST-1) | 0.0286 |
| 9427 (ST-144) vs. 10276 (ST-9) | < 0.0001 |  | 203 (ST-8) vs. 104 (ST-1) | 0.0284 |
| 9427 (ST-144) vs. 211 (ST-28) | 0.0175 |  | 211 (ST-28) vs. 6 (ST-1) | 0.0259 |
| 9427 (ST-144) vs. 7339 (ST-12) | 0.0015 |  | 211 (ST-28) vs. 104 (ST-1) | 0.0257 |
| 9427 (ST-144) vs. 231 (ST-17) | 0.0162 |  | 231 (ST-17) vs. 6 (ST-1) | 0.0199 |
| 9427 (ST-144) vs. 9731 (ST-17) | < 0.0001 |  | 231 (ST-17) vs. 104 (ST-1) | 0.0198 |
| 9427 (ST-144) vs. 8422 (ST-291) | 0.0015 |  | 9731 (ST-17) vs. 123 (ST-498) | 0.0129 |
| 9427 (ST-144) vs. 6 (ST-1) | < 0.0001 |  | 123 (ST-498) vs. 6 (ST-1) | 0.0009 |
| 9427 (ST-144) vs. 104 (ST-1) | < 0.0001 |  | 123 (ST-498) vs. 104 (ST-1) | 0.0004 |

**Table S2: Statistically significant differences in CXCL9 chemokine production 24 hours after macrophage infection with individual GBS isolates,** determined by one-way ANOVA with *post-hoc* Šidák test.

| **GBS isolates** | **Šidák test for multiple comparisons   (p-value)** |  | **GBS isolates** | **Šidák test for multiple comparisons   (p-value)** |
| --- | --- | --- | --- | --- |
| 229 (ST-23) vs. 9427 (ST-144) | < 0.0001 |  | 10276 (ST-9) vs. 9731 (ST-17) | < 0.0001 |
| 229 (ST-23) vs. 203 (ST-8) | 0.0447 |  | 10276 (ST-9) vs. 231 (ST-17) | 0.0254 |
| 229 (ST-23) vs. 10276 (ST-9) | 0.0209 |  | 211 (ST-28) vs. 7339 (ST-12) | < 0.0001 |
| 29 (ST-23) vs. 211 (ST-28) | < 0.0001 |  | 211 (ST-28) vs. 231 (ST-17) | < 0.0001 |
| 229 (ST-23) vs. 9731 (ST-17) | < 0.0001 |  | 211 (ST-28) vs. 8422 (ST-291) | < 0.0001 |
| 9427 (ST-144) vs. 211 (ST-28) | < 0.0001 |  | 211 (ST-28) vs. 6 (ST-1) | < 0.0001 |
| 9427 (ST-144) vs. 7339 (ST-12) | < 0.0001 |  | 211 (ST-28) vs. 104 (ST-1) | < 0.0001 |
| 9427 (ST-144) vs. 231 (ST-17) | < 0.0001 |  | 211 (ST-28) vs. 123 (ST-498) | < 0.0001 |
| 9427 (ST-144) vs. 9731 (ST-17) | < 0.0001 |  | 7339 (ST-12) vs. 9731 (ST-17) | 0.0008 |
| 9427 (ST-144) vs. 6 (ST-1) | 0.0071 |  | 7339 (ST-12) vs. 8422 (ST-291) | 0.0014 |
| 9427 (ST-144) vs. 104 (ST-1) | 0.0011 |  | 7339 (ST-12) vs. 6 (ST-1) | 0.0412 |
| 9427 (ST-144) vs. 123 (ST-498) | 0.0008 |  | 231 (ST-17) vs. 9731 (ST-17) | < 0.0001 |
| 203 (ST-8) vs. 211 (ST-28) | < 0.0001 |  | 9731 (ST-17) vs. 8422 (ST-291) | < 0.0001 |
| 203 (ST-8) vs. 7339 (ST-12) | 0.0011 |  | 9731 (ST-17) vs. 6 (ST-1) | < 0.0001 |
| 203 (ST-8) vs. 9731 (ST-17) | < 0.0001 |  | 9731 (ST-17) vs. 104 (ST-1) | < 0.0001 |
| 10276 (ST-9) vs. 211 (ST-28) | < 0.0001 |  | 9731 (ST-17) vs. 123 (ST-498) | < 0.0001 |
| 10276 (ST-9) vs. 7339 (ST-12) | 0.0004 |  |  |  |

**Table S3: Statistically significant differences in IL-1β production 3 hours after macrophage infection with individual GBS isolates,** determined by one-way ANOVA with *post-hoc* Šidák test.

| **GBS isolates** | **Šidák test for multiple comparisons   (p-value)** |  | **GBS isolates** | **Šidák test for multiple comparisons   (p-value)** |
| --- | --- | --- | --- | --- |
| 229 (ST-23) vs. 203 (ST-8) | 0.0008 |  | 203 (ST-8) vs. 123 (ST-498) | 0.0226 |
| 229 (ST-23) vs. 211 (ST-28) | 0.0019 |  | 10276 (ST-9) vs. 211 (ST-28) | 0.0028 |
| 229 (ST-23) vs. 7339 (ST-12) | 0.0003 |  | 10276 (ST-9) vs. 7339 (ST-12) | 0.0005 |
| 229 (ST-23) vs. 231 (ST-17) | < 0.0001 |  | 10276 (ST-9) vs. 231 (ST-17) | < 0.0001 |
| 229 (ST-23) vs. 9731 (ST-17) | < 0.0001 |  | 10276 (ST-9) vs. 9731 (ST-17) | < 0.0001 |
| 229 (ST-23) vs. 8422 (ST-291) | 0.0082 |  | 10276 (ST-9) vs. 8422 (ST-291) | 0.0115 |
| 229 (ST-23) vs. 104 (ST-1) | 0.0002 |  | 10276 (ST-9) vs. 104 (ST-1) | 0.0003 |
| 9427 (ST-144) vs. 203 (ST-8) | < 0.0001 |  | 211 (ST-28) vs. 123 (ST-498) | 0.0351 |
| 9427 (ST-144) vs. 211 (ST-28) | < 0.0001 |  | 7339 (ST-12) vs. 123 (ST-498) | 0.007 |
| 9427 (ST-144) vs. 7339 (ST-12) | < 0.0001 |  | 231 (ST-17) vs. 6 (ST-1) | 0.0118 |
| 9427 (ST-144) vs. 231 (ST-17) | < 0.0001 |  | 231 (ST-17) vs. 123 (ST-498) | 0.0009 |
| 9427 (ST-144) vs. 9731 (ST-17) | < 0.0001 |  | 9731 (ST-17) vs. 6 (ST-1) | 0.0083 |
| 9427 (ST-144) vs. 8422 (ST-291) | < 0.0001 |  | 9731 (ST-17) vs. 123 (ST-498) | 0.0006 |
| 9427 (ST-144) vs. 6 (ST-1) | 0.0004 |  | 8422 (ST-291) vs. 123 (ST-498) | 0.0493 |
| 9427 (ST-144) vs. 104 (ST-1) | < 0.0001 |  | 104 (ST-1) vs. 6 (ST-1) | 0.0151 |
| 9427 (ST-144) vs. 123 (ST-498) | 0.0061 |  | 104 (ST-1) vs. 123 (ST-498) | 0.0007 |
| 203 (ST-8) vs. 10276 (ST-9) | 0.0014 |  |  |  |

**Table S4: Statistically significant differences in IL-1β production 24 hours after macrophage infection with individual GBS isolates,** determined by one-way ANOVA with *post-hoc* Šidák test.

| **GBS isolates** | **Šidák test for multiple comparisons   (p-value)** |  | **GBS isolates** | **Šidák test for multiple comparisons   (p-value)** |
| --- | --- | --- | --- | --- |
| 229 (ST-23) vs. 7339 (ST-12) | 0.0279 |  | 10276 (ST-9) vs. 7339 (ST-12) | < 0.0001 |
| 229 (ST-23) vs. 6 (ST-1) | < 0.0001 |  | 10276 (ST-9) vs. 9731 (ST-17) | 0.001 |
| 9427 (ST-144) vs. 203 (ST-8) | 0.0074 |  | 10276 (ST-9) vs. 6 (ST-1) | < 0.0001 |
| 9427 (ST-144) vs. 211 (ST-28) | < 0.0001 |  | 211 (ST-28) vs. 123 (ST-498) | 0.0286 |
| 9427 (ST-144) vs. 7339 (ST-12) | < 0.0001 |  | 7339 (ST-12) vs. 231 (ST-17) | 0.0377 |
| 9427 (ST-144) vs. 231 (ST-17) | 0.0301 |  | 7339 (ST-12) vs. 8422 (ST-291) | 0.0043 |
| 9427 (ST-144) vs. 9731 (ST-17) | < 0.0001 |  | 7339 (ST-12) vs. 123 (ST-498) | 0.0019 |
| 9427 (ST-144) vs. 6 (ST-1) | < 0.0001 |  | 231 (ST-17) vs. 6 (ST-1) | 0.0001 |
| 9427 (ST-144) vs. 104 (ST-1) | 0.0065 |  | 9731 (ST-17) vs. 6 (ST-1) | 0.0323 |
| 203 (ST-8) vs. 6 (ST-1) | 0.0012 |  | 9731 (ST-17) vs. 123 (ST-498) | 0.0281 |
| 10276 (ST-9) vs. 203 (ST-8) | 0.024 |  | 8422 (ST-291) vs. 6 (ST-1) | < 0.0001 |
| 203 (ST-8) vs. 6 (ST-1) | 0.0002 |  | 6 (ST-1) vs. 104 (ST-1) | 0.0003 |
| 10276 (ST-9) vs. 211 (ST-28) | 0.0005 |  | 6 (ST-1) vs. 123 (ST-498) | < 0.0001 |

**Table S5: Statistically significant differences in IL-6 production 3 hours after macrophage infection with individual GBS isolates,** determined by one-way ANOVA with *post-hoc* Šidák test.

| **GBS isolates** | **Šidák test for multiple comparisons   (p-value)** |  | **GBS isolates** | **Šidák test for multiple comparisons   (p-value)** |
| --- | --- | --- | --- | --- |
| 229 (ST-23) vs. 9427 (ST-144) | 0.0144 |  | 9427 (ST-144) vs. 104 (ST-1) | 0.0386 |
| 9427 (ST-144) vs. 203 (ST-8) | 0.0004 |  | 203 (ST-8) vs. 123 (ST-498) | 0.0032 |
| 9427 (ST-144) vs. 10276 (ST-9) | 0.0087 |  | 10276 (ST-9) vs. 123 (ST-498) | 0.0432 |
| 9427 (ST-144) vs. 211 (ST-28) | 0.0173 |  | 7339 (ST-12) vs. 123 (ST-498) | 0.0499 |
| 9427 (ST-144) vs. 7339 (ST-12) | 0.0142 |  | 231 (ST-17) vs. 123 (ST-498) | 0.0012 |
| 9427 (ST-144) vs. 231 (ST-17) | 0.0003 |  | 9731 (ST-17) vs. 123 (ST-498) | 0.0002 |
| 9427 (ST-144) vs. 9731 (ST-17) | < 0.0001 |  | 8422 (ST-291) vs. 123 (ST-498) | 0.001 |
| 9427 (ST-144) vs. 8422 (ST-291) | 0.0004 |  | 6 (ST-1) vs. 123 (ST-498) | 0.0001 |
| 9427 (ST-144) vs. 6 (ST-1) | < 0.0001 |  | 104 (ST-1) vs. 123 (ST-498) | 0.0206 |

**Table S6: Statistically significant differences in IL-6 production 24 hours after macrophage infection with individual GBS isolates,** determined by one-way ANOVA with *post-hoc* Šidák test.

| **GBS isolates** | **Šidák test for multiple comparisons   (p-value)** |  | **GBS isolates** | **Šidák test for multiple comparisons   (p-value)** |
| --- | --- | --- | --- | --- |
| 229 (ST-23) vs. 7339 (ST-12) | < 0.0001 |  | 211 (ST-28) vs. 7339 (ST-12) | < 0.0001 |
| 229 (ST-23) vs. 9731 (ST-17) | 0.0207 |  | 7339 (ST-12) vs. 231 (ST-17) | < 0.0001 |
| 9427 (ST-144) vs. 211 (ST-28) | 0.0003 |  | 7339 (ST-12) vs. 9731 (ST-17) | < 0.0001 |
| 9427 (ST-144) vs. 7339 (ST-12) | < 0.0001 |  | 7339 (ST-12) vs. 8422 (ST-291) | < 0.0001 |
| 9427 (ST-144) vs. 231 (ST-17) | < 0.0001 |  | 7339 (ST-12) vs. 6 (ST-1) | < 0.0001 |
| 9427 (ST-144) vs. 9731 (ST-17) | < 0.0001 |  | 7339 (ST-12) vs. 104 (ST-1) | < 0.0001 |
| 9427 (ST-144) vs. 8422 (ST-291) | 0.0437 |  | 7339 (ST-12) vs. 123 (ST-498) | < 0.0001 |
| 9427 (ST-144) vs. 123 (ST-498) | 0.0311 |  | 231 (ST-17) vs. 6 (ST-1) | 0.0157 |
| 203 (ST-8) vs. 7339 (ST-12) | < 0.0001 |  | 231 (ST-17) vs. 104 (ST-1) | 0.0149 |
| 203 (ST-8) vs. 9731 (ST-17) | 0.0162 |  | 9731 (ST-17) vs. 8422 (ST-291) | 0.0204 |
| 10276 (ST-9) vs. 211 (ST-28) | 0.0111 |  | 9731 (ST-17) vs. 6 (ST-1) | 0.002 |
| 10276 (ST-9) vs. 7339 (ST-12) | < 0.0001 |  | 9731 (ST-17) vs. 104 (ST-1) | 0.0019 |
| 10276 (ST-9) vs. 231 (ST-17) | 0.001 |  | 9731 (ST-17) vs. 123 (ST-498) | 0.0285 |
| 10276 (ST-9) vs. 9731 (ST-17) | 0.0001 |  |  |  |

**Table S7: Statistically significant differences in IL-10 production 3 hours after macrophage infection with individual GBS isolates,** determined by one-way ANOVA with *post-hoc* Šidák test.

| **GBS isolates** | **Šidák test for multiple comparisons   (p-value)** |  | **GBS isolates** | **Šidák test for multiple comparisons   (p-value)** |
| --- | --- | --- | --- | --- |
| 229 (ST-23) vs. 6 (ST-1) | 0.0204 |  | 9427 (ST-144) vs. 104 (ST-1) | 0.0206 |
| 9427 (ST-144) vs. 211 (ST-28) | 0.0104 |  | 203 (ST-8) vs. 6 (ST-1) | 0.0286 |
| 9427 (ST-144) vs. 7339 (ST-12) | 0.0002 |  | 10276 (ST-9) vs. 6 (ST-1) | 0.0037 |
| 9427 (ST-144) vs. 231 (ST-17) | 0.0136 |  | 7339 (ST-12) vs. 123 (ST-498) | 0.0481 |
| 9427 (ST-144) vs. 9731 (ST-17) | 0.0019 |  | 6 (ST-1) vs. 104 (ST-1) | 0.0464 |
| 9427 (ST-144) vs. 8422 (ST-291) | 0.0019 |  | 6 (ST-1) vs. 123 (ST-498) | 0.0011 |
| 9427 (ST-144) vs. 6 (ST-1) | < 0.0001 |  |  |  |

**Table S8: Statistically significant differences in IL-10 production 24 hours after macrophage infection with individual GBS isolates,** determined by one-way ANOVA with *post-hoc* Šidák test.

| **GBS isolates** | **Šidák test for multiple comparisons   (p-value)** |  | **GBS isolates** | **Šidák test for multiple comparisons   (p-value)** |
| --- | --- | --- | --- | --- |
| 229 (ST-23) vs. 203 (ST-8) | 0.0342 |  | 203 (ST-8) vs. 123 (ST-498) | 0.0085 |
| 229 (ST-23) vs. 9731 (ST-17) | 0.0322 |  | 10276 (ST-9) vs. 7339 (ST-12) | 0.025 |
| 9427 (ST-144) vs. 203 (ST-8) | 0.0011 |  | 10276 (ST-9) vs. 231 (ST-17) | 0.0388 |
| 9427 (ST-144) vs. 7339 (ST-12) | 0.0169 |  | 10276 (ST-9) vs. 9731 (ST-17) | 0.0016 |
| 9427 (ST-144) vs. 231 (ST-17) | 0.0268 |  | 211 (ST-28) vs. 9731 (ST-17) | 0.0281 |
| 9427 (ST-144) vs. 9731 (ST-17) | 0.001 |  | 7339 (ST-12) vs. 6 (ST-1) | 0.0088 |
| 203 (ST-8) vs. 10276 (ST-9) | 0.002 |  | 231 (ST-17) vs. 6 (ST-1) | 0.0117 |
| 203 (ST-8) vs. 211 (ST-28) | 0.0383 |  | 9731 (ST-17) vs. 6 (ST-1) | 0.0003 |
| 203 (ST-8) vs. 6 (ST-1) | 0.0008 |  | 9731 (ST-17) vs. 104 (ST-1) | 0.0157 |
| 203 (ST-8) vs. 104 (ST-1) | 0.0372 |  | 9731 (ST-17) vs. 123 (ST-498) | 0.0035 |

**Table S9: Statistically significant differences in IL-12p40 production 3 hours after macrophage infection with individual GBS isolates,** determined by one-way ANOVA with *post-hoc* Šidák test.

| **GBS isolates** | **Šidák test for multiple comparisons   (p-value)** |  | **GBS isolates** | **Šidák test for multiple comparisons   (p-value)** |
| --- | --- | --- | --- | --- |
| 229 (ST-23) vs. 9427 (ST-144) | < 0.0001 |  | 203 (ST-8) vs. 123 (ST-498) | < 0.0001 |
| 229 (ST-23) vs. 203 (ST-8) | 0.0004 |  | 10276 (ST-9) vs. 7339 (ST-12) | 0.025 |
| 229 (ST-23) vs. 7339 (ST-12) | 0.0025 |  | 10276 (ST-9) vs. 231 (ST-17) | 0.0002 |
| 229 (ST-23) vs. 231 (ST-17) | 0.0126 |  | 10276 (ST-9) vs. 8422 (ST-291) | 0.0232 |
| 229 (ST-23) vs. 6 (ST-1) | 0.0007 |  | 10276 (ST-9) vs. 6 (ST-1) | 0.0078 |
| 229 (ST-23) vs. 104 (ST-1) | 0.001 |  | 10276 (ST-9) vs. 104 (ST-1) | 0.0109 |
| 229 (ST-23) vs. 123 (ST-498) | 0.0055 |  | 211 (ST-28) vs. 7339 (ST-12) | 0.0026 |
| 9427 (ST-144) vs. 203 (ST-8) | 0.0256 |  | 211 (ST-28) vs. 231 (ST-17) | 0.0016 |
| 9427 (ST-144) vs. 10276 (ST-9) | < 0.0001 |  | 211 (ST-28) vs. 6 (ST-1) | 0.0007 |
| 9427 (ST-144) vs. 211 (ST-28) | < 0.0001 |  | 211 (ST-28) vs. 104 (ST-1) | 0.001 |
| 9427 (ST-144) vs. 7339 (ST-12) | < 0.0001 |  | 211 (ST-28) vs. 123 (ST-498) | 0.0058 |
| 9427 (ST-144) vs. 231 (ST-17) | 0.0008 |  | 7339 (ST-12) vs. 231 (ST-17) | < 0.0001 |
| 9427 (ST-144) vs. 9731 (ST-17) | < 0.0001 |  | 7339 (ST-12) vs. 8422 (ST-291) | < 0.0001 |
| 9427 (ST-144) vs. 8422 (ST-291) | < 0.0001 |  | 231 (ST-17) vs. 9731 (ST-17) | < 0.0001 |
| 9427 (ST-144) vs. 6 (ST-1) | < 0.0001 |  | 231 (ST-17) vs. 6 (ST-1) | < 0.0001 |
| 9427 (ST-144) vs. 104 (ST-1) | < 0.0001 |  | 231 (ST-17) vs. 104 (ST-1) | < 0.0001 |
| 9427 (ST-144) vs. 123 (ST-498) | < 0.0001 |  | 231 (ST-17) vs. 123 (ST-498) | < 0.0001 |
| 203 (ST-8) vs. 10276 (ST-9) | < 0.0001 |  | 9731 (ST-17) vs. 8422 (ST-291) | 0.0018 |
| 203 (ST-8) vs. 211 (ST-28) | < 0.0001 |  | 9731 (ST-17) vs. 6 (ST-1) | 0.0233 |
| 203 (ST-8) vs. 7339 (ST-12) | < 0.0001 |  | 9731 (ST-17) vs. 104 (ST-1) | 0.0312 |
| 203 (ST-8) vs. 9731 (ST-17) | < 0.0001 |  | 8422 (ST-291) vs. 6 (ST-1) | < 0.0001 |
| 203 (ST-8) vs. 6 (ST-1) | < 0.0001 |  | 8422 (ST-291) vs. 104 (ST-1) | < 0.0001 |
| 203 (ST-8) vs. 104 (ST-1) | < 0.0001 |  | 8422 (ST-291) vs. 123 (ST-498) | < 0.0001 |

**Table S10: Statistically significant differences in IL-12p40 production 24 hours after macrophage infection with individual GBS isolates,** determined by one-way ANOVA with *post-hoc* Šidák test.

| **GBS isolates** | **Šidák test for multiple comparisons   (p-value)** |  | **GBS isolates** | **Šidák test for multiple comparisons   (p-value)** |
| --- | --- | --- | --- | --- |
| 229 (ST-23) vs. 211 (ST-28) | 0.0054 |  | 211 (ST-28) vs. 8422 (ST-291) | 0.0243 |
| 229 (ST-23) vs. 7339 (ST-12) | 0.0006 |  | 211 (ST-28) vs. 6 (ST-1) | 0.0004 |
| 229 (ST-23) vs. 231 (ST-17) | 0.0004 |  | 211 (ST-28) vs. 104 (ST-1) | 0.0011 |
| 229 (ST-23) vs. 9731 (ST-17) | 0.0011 |  | 211 (ST-28) vs. 123 (ST-498) | 0.0023 |
| 9427 (ST-144) vs. 211 (ST-28) | < 0.0001 |  | 7339 (ST-12) vs. 8422 (ST-291) | 0.0028 |
| 9427 (ST-144) vs. 7339 (ST-12) | < 0.0001 |  | 7339 (ST-12) vs. 6 (ST-1) | < 0.0001 |
| 9427 (ST-144) vs. 231 (ST-17) | < 0.0001 |  | 7339 (ST-12) vs. 104 (ST-1) | < 0.0001 |
| 9427 (ST-144) vs. 9731 (ST-17) | < 0.0001 |  | 7339 (ST-12) vs. 123 (ST-498) | 0.0002 |
| 9427 (ST-144) vs. 8422 (ST-291) | 0.0249 |  | 231 (ST-17) vs. 8422 (ST-291) | 0.0018 |
| 203 (ST-8) vs. 211 (ST-28) | 0.0047 |  | 231 (ST-17) vs. 6 (ST-1) | < 0.0001 |
| 203 (ST-8) vs. 7339 (ST-12) | 0.0005 |  | 231 (ST-17) vs. 104 (ST-1) | < 0.0001 |
| 203 (ST-8) vs. 231 (ST-17) | 0.0004 |  | 231 (ST-17) vs. 123 (ST-498) | 0.0001 |
| 203 (ST-8) vs. 9731 (ST-17) | 0.001 |  | 9731 (ST-17) vs. 8422 (ST-291) | 0.0034 |
| 10276 (ST-9) vs. 211 (ST-28) | < 0.0001 |  | 9731 (ST-17) vs. 6 (ST-1) | < 0.0001 |
| 10276 (ST-9) vs. 7339 (ST-12) | < 0.0001 |  | 9731 (ST-17) vs. 104 (ST-1) | 0.0001 |
| 10276 (ST-9) vs. 231 (ST-17) | < 0.0001 |  | 9731 (ST-17) vs. 123 (ST-498) | 0.0003 |
| 10276 (ST-9) vs. 9731 (ST-17) | < 0.0001 |  |  |  |

**Table S11: Statistically significant differences in CXCL8 production 3 hours after macrophage infection with individual GBS isolates,** determined by one-way ANOVA with *post-hoc* Šidák test.

| **GBS isolates** | **Šidák test for multiple comparisons   (p-value)** |  | **GBS isolates** | **Šidák test for multiple comparisons   (p-value)** |
| --- | --- | --- | --- | --- |
| 229 (ST-23) vs. 8422 (ST-291) | 0.0316 |  | 211 (ST-28) vs. 104 (ST-1) | < 0.0001 |
| 229 (ST-23) vs. 104 (ST-1) | < 0.0001 |  | 211 (ST-28) vs. 123 (ST-498) | < 0.0001 |
| 229 (ST-23) vs. 123 (ST-498) | 0.0004 |  | 7339 (ST-12) vs. 104 (ST-1) | < 0.0001 |
| 9427 (ST-144) vs. 231 (ST-17) | 0.0172 |  | 7339 (ST-12) vs. 123 (ST-498) | < 0.0001 |
| 9427 (ST-144) vs. 9731 (ST-17) | 0.0297 |  | 231 (ST-17) vs. 104 (ST-1) | < 0.0001 |
| 9427 (ST-144) vs. 8422 (ST-291) | 0.0007 |  | 231 (ST-17) vs. 123 (ST-498) | < 0.0001 |
| 9427 (ST-144) vs. 6 (ST-1) | 0.0073 |  | 9731 (ST-17) vs. 104 (ST-1) | < 0.0001 |
| 9427 (ST-144) vs. 104 (ST-1) | < 0.0001 |  | 9731 (ST-17) vs. 123 (ST-498) | < 0.0001 |
| 9427 (ST-144) vs. 123 (ST-498) | 0.0172 |  | 8422 (ST-291) vs. 104 (ST-1) | < 0.0001 |
| 203 (ST-8) vs. 104 (ST-1) | < 0.0001 |  | 8422 (ST-291) vs. 123 (ST-498) | < 0.0001 |
| 203 (ST-8) vs. 123 (ST-498) | < 0.0001 |  | 6 (ST-1) vs. 104 (ST-1) | < 0.0001 |
| 10276 (ST-9) vs. 104 (ST-1) | < 0.0001 |  | 6 (ST-1) vs. 123 (ST-498) | < 0.0001 |
| 10276 (ST-9) vs. 123 (ST-498) | < 0.0001 |  | 104 (ST-1) vs. 123 (ST-498) | 0.0017 |

**Table S12: Statistically significant differences in CXCL8 production 24 hours after macrophage infection with individual GBS isolates,** determined by one-way ANOVA with *post-hoc* Šidák test.

| **GBS isolates** | **Šidák test for multiple comparisons   (p-value)** |
| --- | --- |
| 203 (ST-8) vs. 104 (ST-1) | 0.013 |
| 231 (ST-17) vs. 104 (ST-1) | 0.0042 |
| 8422 (ST-291) vs. 104 (ST-1) | 0.001 |
| 8422 (ST-291) vs. 123 (ST-498) | 0.0378 |

**Table S13: Statistically significant differences in CCL8 production 3 hours after macrophage infection with individual GBS isolates,** determined by one-way ANOVA with *post-hoc* Šidák test.

| **GBS isolates** | **Šidák test for multiple comparisons   (p-value)** |  | **GBS isolates** | **Šidák test for multiple comparisons   (p-value)** |
| --- | --- | --- | --- | --- |
| 229 (ST-23) vs. 211 (ST-28) | < 0.0001 |  | 211 (ST-28) vs. 6 (ST-1) | 0.0019 |
| 229 (ST-23) vs. 7339 (ST-12) | < 0.0001 |  | 211 (ST-28) vs. 104 (ST-1) | < 0.0001 |
| 229 (ST-23) vs. 123 (ST-498) | 0.0042 |  | 7339 (ST-12) vs. 231 (ST-17) | 0.0006 |
| 9427 (ST-144) vs. 211 (ST-28) | 0.0053 |  | 7339 (ST-12) vs. 9731 (ST-17) | < 0.0001 |
| 9427 (ST-144) vs. 7339 (ST-12) | 0.0003 |  | 7339 (ST-12) vs. 8422 (ST-291) | 0.017 |
| 203 (ST-8) vs. 211 (ST-28) | 0.0008 |  | 7339 (ST-12) vs. 6 (ST-1) | < 0.0001 |
| 203 (ST-8) vs. 7339 (ST-12) | < 0.0001 |  | 7339 (ST-12) vs. 104 (ST-1) | < 0.0001 |
| 203 (ST-8) vs. 123 (ST-498) | 0.0351 |  | 9731 (ST-17) vs. 8422 (ST-291) | 0.007 |
| 10276 (ST-9) vs. 211 (ST-28) | < 0.0001 |  | 9731 (ST-17) vs. 123 (ST-498) | 0.0003 |
| 10276 (ST-9) vs. 7339 (ST-12) | < 0.0001 |  | 8422 (ST-291) vs. 104 (ST-1) | 0.039 |
| 10276 (ST-9) vs. 123 (ST-498) | 0.0028 |  | 6 (ST-1) vs. 123 (ST-498) | 0.0206 |
| 211 (ST-28) vs. 231 (ST-17) | 0.0119 |  | 104 (ST-1) vs. 123 (ST-498) | 0.0007 |
| 211 (ST-28) vs. 9731 (ST-17) | < 0.0001 |  |  |  |

**Table S14: Statistically significant differences in CCL8 production 24 hours after macrophage infection with individual GBS isolates,** determined by one-way ANOVA with *post-hoc* Šidák test.

| **GBS isolates** | **Šidák test for multiple comparisons   (p-value)** |  | **GBS isolates** | **Šidák test for multiple comparisons   (p-value)** |
| --- | --- | --- | --- | --- |
| 229 (ST-23) vs. 104 (ST-1) | 0.0388 |  | 231 (ST-17) vs. 104 (ST-1) | 0.0123 |
| 9427 (ST-144) vs. 104 (ST-1) | 0.0235 |  | 9731 (ST-17) vs. 104 (ST-1) | 0.0135 |
| 203 (ST-8) vs. 104 (ST-1) | 0.0221 |  | 8422 (ST-291) vs. 104 (ST-1) | 0.0072 |
| 10276 (ST-9) vs. 104 (ST-1) | 0.0235 |  | 6 (ST-1) vs. 104 (ST-1) | 0.0051 |
| 211 (ST-28) vs. 104 (ST-1) | 0.0225 |  | 123 (ST-498) vs. 104 (ST-1) | 0.0228 |

**Table S15: Statistically significant differences in IL-18 production 3 hours after macrophage infection with individual GBS isolates,** determined by one-way ANOVA with *post-hoc* Šidák test.

| **GBS isolates** | **Šidák test for multiple comparisons   (p-value)** |  | **GBS isolates** | **Šidák test for multiple comparisons   (p-value)** |
| --- | --- | --- | --- | --- |
| 229 (ST-23) vs. 211 (ST-28) | 0.0092 |  | 9427 (ST-144) vs. 123 (ST-498) | 0.0372 |
| 229 (ST-23) vs. 7339 (ST-12) | 0.0105 |  | 203 (ST-8) vs. 10276 (ST-9) | 0.023 |
| 229 (ST-23) vs. 231 (ST-17) | 0.0023 |  | 10276 (ST-9) vs. 211 (ST-28) | 0.0017 |
| 229 (ST-23) vs. 9731 (ST-17) | 0.0019 |  | 10276 (ST-9) vs. 7339 (ST-12) | 0.0019 |
| 229 (ST-23) vs. 104 (ST-1) | 0.0026 |  | 10276 (ST-9) vs. 231 (ST-17) | 0.0004 |
| 9427 (ST-144) vs. 203 (ST-8) | < 0.0001 |  | 10276 (ST-9) vs. 9731 (ST-17) | 0.0003 |
| 9427 (ST-144) vs. 211 (ST-28) | < 0.0001 |  | 10276 (ST-9) vs. 104 (ST-1) | 0.0004 |
| 9427 (ST-144) vs. 7339 (ST-12) | < 0.0001 |  | 211 (ST-28) vs. 123 (ST-498) | 0.0045 |
| 9427 (ST-144) vs. 231 (ST-17) | < 0.0001 |  | 7339 (ST-12) vs. 123 (ST-498) | 0.0045 |
| 9427 (ST-144) vs. 9731 (ST-17) | < 0.0001 |  | 231 (ST-17) vs. 123 (ST-498) | 0.0007 |
| 9427 (ST-144) vs. 8422 (ST-291) | < 0.0001 |  | 9731 (ST-17) vs. 123 (ST-498) | 0.0005 |
| 9427 (ST-144) vs. 6 (ST-1) | < 0.0001 |  | 6 (ST-1) vs. 123 (ST-498) | 0.0475 |
| 9427 (ST-144) vs. 104 (ST-1) | < 0.0001 |  | 104 (ST-1) vs. 123 (ST-498) | 0.0002 |

**Table S16: Statistically significant differences in IL-18 production 24 hours after macrophage infection with individual GBS isolates,** determined by one-way ANOVA with *post-hoc* Šidák test.

| **GBS isolates** | **Šidák test for multiple comparisons   (p-value)** |  | **GBS isolates** | **Šidák test for multiple comparisons   (p-value)** |
| --- | --- | --- | --- | --- |
| 9427 (ST-144) vs. 203 (ST-8) | 0.0033 |  | 231 (ST-17) vs. 104 (ST-1) | 0.0052 |
| 9427 (ST-144) vs. 8422 (ST-291) | 0.0027 |  | 9731 (ST-17) vs. 8422 (ST-291) | 0.0248 |
| 203 (ST-8) vs. 10276 (ST-9) | 0.0385 |  | 8422 (ST-291) vs. 104 (ST-1) | < 0.0001 |
| 203 (ST-8) vs. 104 (ST-1) | < 0.0001 |  | 8422 (ST-291) vs. 123 (ST-498) | 0.0214 |
| 10276 (ST-9) vs. 8422 (ST-291) | 0.0293 |  | 6 (ST-1) vs. 104 (ST-1) | 0.0004 |
| 211 (ST-28) vs. 104 (ST-1) | 0.0429 |  | 104 (ST-1) vs. 123 (ST-498) | 0.015 |
| 7339 (ST-12) vs. 8422 (ST-291) | 0.0472 |  |  |  |

**Table S17: Statistically significant differences in TNF-α production 3 hours after macrophage infection with individual GBS isolates,** determined by one-way ANOVA with *post-hoc* Šidák test.

| **GBS isolates** | **Šidák test for multiple comparisons   (p-value)** |  | **GBS isolates** | **Šidák test for multiple comparisons   (p-value)** |
| --- | --- | --- | --- | --- |
| 229 (ST-23) vs. 123 (ST-498) | 0.0002 |  | 231 (ST-17) vs. 123 (ST-498) | < 0.0001 |
| 9427 (ST-144) vs. 123 (ST-498) | 0.0003 |  | 9731 (ST-17) vs. 123 (ST-498) | < 0.0001 |
| 203 (ST-8) vs. 123 (ST-498) | < 0.0001 |  | 8422 (ST-291) vs. 123 (ST-498) | < 0.0001 |
| 10276 (ST-9) vs. 123 (ST-498) | 0.0001 |  | 6 (ST-1) vs. 123 (ST-498) | < 0.0001 |
| 211 (ST-28) vs. 123 (ST-498) | 0.0002 |  | 104 (ST-1) vs. 123 (ST-498) | < 0.0001 |
| 7339 (ST-12) vs. 123 (ST-498) | 0.0002 |  |  |  |

**Table S18: Statistically significant differences in TNF-α production 24 hours after macrophage infection with individual GBS isolates,** determined by one-way ANOVA with *post-hoc* Šidák test.

| **GBS isolates** | **Šidák test for multiple comparisons   (p-value)** |  | **GBS isolates** | **Šidák test for multiple comparisons   (p-value)** |
| --- | --- | --- | --- | --- |
| 229 (ST-23) vs. 7339 (ST-12) | < 0.0001 |  | 10276 (ST-9) vs. 231 (ST-17) | 0.0411 |
| 9427 (ST-144) vs. 211 (ST-28) | 0.0199 |  | 10276 (ST-9) vs. 9731 (ST-17) | 0.0066 |
| 9427 (ST-144) vs. 7339 (ST-12) | < 0.0001 |  | 211 (ST-28) vs. 7339 (ST-12) | 0.0018 |
| 9427 (ST-144) vs. 231 (ST-17) | 0.0059 |  | 7339 (ST-12) vs. 231 (ST-17) | 0.0054 |
| 9427 (ST-144) vs. 9731 (ST-17) | 0.0008 |  | 7339 (ST-12) vs. 9731 (ST-17) | 0.0332 |
| 9427 (ST-144) vs. 6 (ST-1) | 0.0443 |  | 7339 (ST-12) vs. 8422 (ST-291) | < 0.0001 |
| 9427 (ST-144) vs. 123 (ST-498) | 0.0369 |  | 7339 (ST-12) vs. 6 (ST-1) | 0.0006 |
| 203 (ST-8) vs. 7339 (ST-12) | < 0.0001 |  | 7339 (ST-12) vs. 104 (ST-1) | < 0.0001 |
| 10276 (ST-9) vs. 7339 (ST-12) | < 0.0001 |  | 7339 (ST-12) vs. 123 (ST-498) | 0.0007 |

**Table S19: Statistically significant differences in CXCL9 chemokine production according to the specimen**, determined by Kruskal–Wallis test with *post hoc* Dunn’s multiple comparison test.

| **GBS isolates** | **Dunn's test for multiple comparisons   (p-value)** |  | **GBS isolates** | **Dunn's test for multiple comparisons   (p-value)** |
| --- | --- | --- | --- | --- |
| CSF, 24h vs. CSF, 3h | < 0.0001 |  | V/V-R, 24h vs. Blood, 3h | < 0.0001 |
| Blood, 24h vs. CSF, 3h | < 0.0001 |  | CSF, 24h vs. V/V-R, 3h | < 0.0001 |
| V/V-R, 24h vs. CSF, 3h | < 0.0001 |  | Blood, 24h vs. V-V/R, 3h | < 0.0001 |
| CSF, 24h vs. Blood, 3h | < 0.0001 |  | V-V/R, 24h vs. V-V/R, 3h | < 0.0001 |
| Blood, 24h vs. Blood, 3h | < 0.0001 |  |  |  |

**Table S20: Statistically significant differences in IL-1β production according to the specimen**, determined by Kruskal–Wallis test with *post hoc* Dunn’s multiple comparison test.

| **GBS isolates** | **Dunn's test for multiple comparisons   (p-value)** |  | **GBS isolates** | **Dunn's test for multiple comparisons   (p-value)** |
| --- | --- | --- | --- | --- |
| Blood, 3h vs. CSF, 3h | 0.0079 |  | V-V/R, 24h vs. Blood, 3h | < 0.0001 |
| CSF, 24h vs. Blood, 3h | < 0.0001 |  | CSF, 24h vs. V/V-R, 3h | 0.0025 |
| Blood, 24h vs. Blood, 3h | < 0.0001 |  | Blood, 24h vs. V/V-R, 3h | 0.0065 |

**Table S21: Statistically significant differences in IL-6 production according to the specimen**, determined by Kruskal–Wallis test with *post hoc* Dunn’s multiple comparison test.

| **GBS isolates** | **Dunn's test for multiple comparisons   (p-value)** |  | **GBS isolates** | **Dunn's test for multiple comparisons   (p-value)** |
| --- | --- | --- | --- | --- |
| CSF, 24h vs. CSF, 3h | 0.0002 |  | V/V-R, 24h vs. Blood, 3h | < 0.0001 |
| Blood, 24h vs. CSF, 3h | < 0.0001 |  | CSF, 24h vs. V/V-R, 3h | 0.0001 |
| V/V-R, 24h vs. CSF, 3h | < 0.0001 |  | Blood, 24h vs. V-V/R, 3h | < 0.0001 |
| CSF, 24h vs. Blood, 3h | 0.0003 |  | V-V/R, 24h vs. V-V/R, 3h | < 0.0001 |
| Blood, 24h vs. Blood, 3h | < 0.0001 |  |  |  |

**Table S22: Statistically significant differences in IL-10 production according to the specimen**, determined by Kruskal–Wallis test with *post hoc* Dunn’s multiple comparison test.

| **GBS isolates** | **Dunn's test for multiple comparisons   (p-value)** |  | **GBS isolates** | **Dunn's test for multiple comparisons   (p-value)** |
| --- | --- | --- | --- | --- |
| CSF, 24h vs. CSF, 3h | 0.0008 |  | V/V-R, 24h vs. Blood, 3h | < 0.0001 |
| Blood, 24h vs. CSF, 3h | < 0.0001 |  | CSF 24h vs. V/V-R, 3h | 0.0007 |
| V/V-R, 24h vs. CSF, 3h | < 0.0001 |  | Blood, 24h vs. V-V/R, 3h | < 0.0001 |
| CSF, 24h vs. Blood, 3h | 0.0048 |  | V-V/R, 24h vs. V-V/R, 3h | < 0.0001 |
| Blood, 24h vs. Blood, 3h | < 0.0001 |  |  |  |

**Table S23: Statistically significant differences in IL-12p40 production according to the specimen**, determined by Kruskal–Wallis test with *post hoc* Dunn’s multiple comparison test.

| **GBS isolates** | **Dunn's test for multiple comparisons   (p-value)** |  | **GBS isolates** | **Dunn's test for multiple comparisons   (p-value)** |
| --- | --- | --- | --- | --- |
| CSF, 24h vs. CSF, 3h | 0.0002 |  | V/V-R, 24h vs. Blood, 3h | < 0.0001 |
| Blood, 24h vs. CSF, 3h | < 0.0001 |  | CSF, 24h vs. V/V-R, 3h | 0.0004 |
| V/V-R, 24h vs. CSF, 3h | < 0.0001 |  | Blood, 24h vs. V-V/R, 3h | < 0.0001 |
| CSF, 24h vs. Blood, 3h | 0.0002 |  | V-V/R, 24h vs. V-V/R, 3h | < 0.0001 |
| Blood, 24h vs. Blood, 3h | < 0.0001 |  |  |  |

**Table S24: Statistically significant differences in CXCL8 chemokine production according to the specimen**, determined by Kruskal–Wallis test with *post hoc* Dunn’s multiple comparison test.

| **GBS isolates** | **Dunn's test for multiple comparisons   (p-value)** |  | **GBS isolates** | **Dunn's test for multiple comparisons   (p-value)** |
| --- | --- | --- | --- | --- |
| CSF, 24h vs. CSF, 3h | < 0.0001 |  | V/V-R, 24h vs. Blood, 3h | < 0.0001 |
| Blood, 24h vs. CSF, 3h | < 0.0001 |  | CSF, 24h vs. V/V-R, 3h | <0.0001 |
| V/V-R, 24h vs. CSF, 3h | < 0.0001 |  | Blood, 24h vs. V-V/R, 3h | < 0.0001 |
| CSF, 24h vs. Blood, 3h | < 0.0001 |  | V-V/R, 24h vs. V-V/R, 3h | < 0.0001 |
| Blood, 24h vs. Blood, 3h | < 0.0001 |  |  |  |

**Table S25: Statistically significant differences in CCL8 chemokine production according to the specimen**, determined by Kruskal–Wallis test with *post hoc* Dunn’s multiple comparison test.

| **GBS isolates** | **Dunn's test for multiple comparisons   (p-value)** |  | **GBS isolates** | **Dunn's test for multiple comparisons   (p-value)** |
| --- | --- | --- | --- | --- |
| CSF, 24h vs. CSF, 3h | < 0.0001 |  | V/V-R, 24h vs. Blood, 3h | < 0.0001 |
| Blood, 24h vs. CSF, 3h | < 0.0001 |  | CSF, 24h vs. V/V-R, 3h | <0.0001 |
| V/V-R, 24h vs. CSF, 3h | < 0.0001 |  | Blood, 24h vs. V-V/R, 3h | < 0.0001 |
| CSF, 24h vs. Blood, 3h | < 0.0001 |  | V-V/R, 24h vs. V-V/R, 3h | < 0.0001 |
| Blood, 24h vs. Blood, 3h | < 0.0001 |  |  |  |

**Table S26: Statistically significant differences in IL-18 production according to the specimen**, determined by Kruskal–Wallis test with *post hoc* Dunn’s multiple comparison test.

| **GBS isolates** | **Dunn's test for multiple comparisons   (p-value)** |
| --- | --- |
| Blood, 3h vs. CSF, 3h | 0.0144 |
| Blood, 24h vs. CSF, 3h | 0.019 |
| V/V-R, 24h vs. CSF, 3h | 0.0255 |

**Table S27: Statistically significant differences in TNF-α production according to the specimen**, determined by Kruskal–Wallis test with *post hoc* Dunn’s multiple comparison test.

| **GBS isolates** | **Dunn's test for multiple comparisons   (p-value)** |  | **GBS isolates** | **Dunn's test for multiple comparisons   (p-value)** |
| --- | --- | --- | --- | --- |
| CSF, 24h vs. CSF, 3h | 0.0327 |  | CSF, 24h vs. V/V-R, 3h | 0.0017 |
| Blood, 24h vs. CSF, 3h | 0.0005 |  | Blood, 24h vs. V-V/R, 3h | < 0.0001 |
| V/V-R, 24h vs. CSF, 3h | 0.0446 |  | V-V/R, 24h vs. V-V/R, 3h | 0.0004 |
| Blood, 24h vs. Blood, 3h | 0.0017 |  |  |  |

**Table S28: Statistically significant differences in *IL1B* gene expression 4 hours after macrophage infection with individual GBS isolates**, determined by one-way ANOVA with *post-hoc* Šidák’s test.

| **GBS isolates** | **Šidák test for multiple comparisons   (p-value)** |
| --- | --- |
| LPS vs. 211 (ST-28) | 0.0104 |
| 211 (ST-28) vs. 7339 (ST-12) | 0.0315 |
| 211 (ST-28) vs. 6 (ST-1) | 0.0408 |

**Table S29: Statistically significant differences in *IL1B* gene expression 24 hours after macrophage infection with individual GBS isolates**, determined by one-way ANOVA with *post-hoc* Šidák’s test.

| **GBS isolates** | **Šidák test for multiple comparisons   (p-value)** |  | **GBS isolates** | **Šidák test for multiple comparisons   (p-value)** |
| --- | --- | --- | --- | --- |
| LPS vs. 9427 (ST-144) | < 0.0001 |  | 9427 (ST-144) vs. 10276 (ST-9) | 0.0088 |
| LPS vs. 203 (ST-8) | < 0.0001 |  | 9427 (ST-144) vs. 104 (ST-1) | 0.0002 |
| LPS vs. 211 (ST-28) | 0.0105 |  | 203 (ST-8) vs. 10276 (ST-9) | 0.0049 |
| LPS vs. 7339 (ST-12) | < 0.0001 |  | 203 (ST-8) vs. 211 (ST-28) | 0.03 |
| LPS vs. 231 (ST-17) | 0.0064 |  | 203 (ST-8) vs. 231 (ST-17) | 0.0477 |
| LPS vs. 9731 (ST-17) | 0.0004 |  | 203 (ST-8) vs. 104 (ST-1) | < 0.0001 |
| LPS vs. 8422 (ST-291) | 0.0007 |  | 203 (ST-8) vs. 123 (ST-498) | 0.0131 |
| LPS vs. 6 (ST-1) | 0.0031 |  | 10276 (ST-9) vs. 7339 (ST-12) | 0.0123 |
| LPS vs. 123 (ST-498) | 0.0244 |  | 7339 (ST-12) vs. 104 (ST-1) | 0.0002 |
| 229 (ST-23) vs. 9427 (ST-144) | 0.0084 |  | 7339 (ST-12) vs. 123 (ST-498) | 0.0241 |
| 229 (ST-23) vs. 203 (ST-8) | 0.0052 |  | 9731 (ST-17) vs. 104 (ST-1) | 0.0066 |
| 229 (ST-23) vs. 7339 (ST-12) | 0.0147 |  | 8422 (ST-291) vs. 104 (ST-1) | 0.0095 |
| 9427 (ST-144) vs. 123 (ST-498) | 0.0233 |  | 6 (ST-1) vs. 104 (ST-1) | 0.0232 |

**Table S30: Statistically significant differences in *IL10* gene expression 4 hours after macrophage infection with individual GBS isolates**, determined by one-way ANOVA with *post-hoc* Šidák’s test.

| **GBS isolates** | **Šidák test for multiple comparisons   (p-value)** |  | **GBS isolates** | **Šidák test for multiple comparisons   (p-value)** |
| --- | --- | --- | --- | --- |
| LPS vs. 6 (ST-1) | 0.0002 |  | 7339 (ST-12)vs. 6 (ST-1) | 0.0004 |
| 229 (ST-23) vs. 6 (ST-1) | 0.0007 |  | 231 (ST-17) vs. 6 (ST-1) | 0.0002 |
| 9427 (ST-144) vs. 6 (ST-1) | 0.0375 |  | 9731 (ST-17) vs. 6 (ST-1) | < 0.0001 |
| 203 (ST-8) vs. 6 (ST-1) | 0.0034 |  | 8422 (ST-291) vs. 6 (ST-1) | 0.0006 |
| 10276 (ST-9) vs. 6 (ST-1) | < 0.0001 |  | 104 (ST-1) vs. 6 (ST-1) | 0.0001 |
| 211 (ST-28) vs. 6 (ST-1) | 0.0042 |  | 123 (ST-498) vs. 6 (ST-1) | 0.0003 |

**Table S31: Statistically significant differences in *IL10* gene expression 24 hours after macrophage infection with individual GBS isolates**, determined by one-way ANOVA with *post-hoc* Šidák’s test.

| **GBS isolates** | **Šidák test for multiple comparisons   (p-value)** |  |
| --- | --- | --- |
| LPS vs. 231 (ST-17) | 0.0261 |  |
| LPS vs. 8422 (ST-291) | 0.0103 |  |
| LPS vs. 6 (ST-1) | 0.0479 |  |

**Table S32: Statistically significant differences in *ACOD1* gene expression 4 hours after macrophage infection with individual GBS isolates**, determined by one-way ANOVA with *post-hoc* Šidák’s test.

| **GBS isolates** | **Šidák test for multiple comparisons   (p-value)** |  | **GBS isolates** | **Šidák test for multiple comparisons   (p-value)** |
| --- | --- | --- | --- | --- |
| LPS vs. 229 (ST-23) | 0.002 |  | LPS vs. 104 (ST-1) | 0.0098 |
| LPS vs. 9427 (ST-144) | 0.0007 |  | LPS vs. 123 (ST-498) | 0.0011 |
| LPS vs. 203 (ST-8) | 0.0013 |  | 211 (ST-28) vs. 229 (ST-23) | 0.0414 |
| LPS vs. 10276 (ST-9) | 0.0006 |  | 211 (ST-28) vs. 9427 (ST-144) | 0.0148 |
| LPS vs. 7339 (ST-12) | 0.0043 |  | 211 (ST-28) vs. 203 (ST-8) | 0.0222 |
| LPS vs. 231 (ST-17) | 0.0046 |  | 211 (ST-28) vs. 10276 (ST-9) | 0.0092 |
| LPS vs. 9731 (ST-17) | 0.0045 |  | 211 (ST-28) vs. 6 (ST-1) | 0.0199 |
| LPS vs. 6 (ST-1) | 0.0015 |  | 211 (ST-28) vs. 123 (ST-498) | 0.0155 |

**Table S33: Statistically significant differences in *ACOD1* gene expression 24 hours after macrophage infection with individual GBS isolates**, determined by one-way ANOVA with *post-hoc* Šidák’s test.

| **GBS isolates** | **Šidák test for multiple comparisons   (p-value)** |  | **GBS isolates** | **Šidák test for multiple comparisons   (p-value)** |
| --- | --- | --- | --- | --- |
| LPS vs. 211 (ST-28) | 0.0135 |  | 7339 (ST-12) vs. 231 (ST-17) | < 0.0001 |
| LPS vs. 231 (ST-17) | < 0.0001 |  | 7339 (ST-12) vs. 9731 (ST-17) | 0.0124 |
| LPS vs. 9731 (ST-17) | 0.0016 |  | 7339 (ST-12) vs. 104 (ST-1) | 0.0219 |
| LPS vs. 104 (ST-1) | 0.0029 |  | 231 (ST-17) vs. 9731 (ST-17) | < 0.0001 |
| 229 (ST-23) vs. 231 (ST-17) | < 0.0001 |  | 231 (ST-17) vs. 8422 (ST-291) | < 0.0001 |
| 9427 (ST-144) vs. 231 (ST-17) | < 0.0001 |  | 231 (ST-17) vs. 6 (ST-1) | < 0.0001 |
| 203 (ST-8) vs. 231 (ST-17) | < 0.0001 |  | 231 (ST-17) vs. 104 (ST-1) | < 0.0001 |
| 10276 (ST-9) vs. 211 (ST-28) | 0.0237 |  | 231 (ST-17) vs. 123 (ST-498) | < 0.0001 |
| 10276 (ST-9) vs. 231 (ST-17) | < 0.0001 |  | 9731 (ST-17) vs. 6 (ST-1) | 0.0013 |
| 10276 (ST-9) vs. 9731 (ST-17) | 0.0029 |  | 9731 (ST-17) vs. 123 (ST-498) | 0.0093 |
| 10276 (ST-9) vs. 104 (ST-1) | 0.0052 |  | 6 (ST-1) vs. 104 (ST-1) | 0.0011 |
| 211 (ST-28) vs. 231 (ST-17) | < 0.0001 |  | 104 (ST-1) vs. 123 (ST-498) | 0.0041 |
| 211 (ST-28) vs. 6 (ST-1) | 0.0182 |  |  |  |

**Table S34: Statistically significant differences in *HIF1A* gene expression 24 hours after macrophage infection with individual GBS isolates**, determined by one-way ANOVA with *post-hoc* Šidák’s test.

| **GBS isolates** | **Šidák test for multiple comparisons   (p-value)** |  | **GBS isolates** | **Šidák test for multiple comparisons   (p-value)** |
| --- | --- | --- | --- | --- |
| LPS vs. 229 (ST-23) | 0.0044 |  | LPS vs. 6 (ST-1) | 0.0062 |
| LPS vs. 9427 (ST-144) | < 0.0001 |  | 9427 (ST-144) vs. 10276 (ST-9) | 0.0013 |
| LPS vs. 203 (ST-8) | 0.002 |  | 9427 (ST-144) vs. 7339 (ST-12) | 0.0018 |
| LPS vs. 211 (ST-28) | 0.0268 |  | 9427 (ST-144) vs. 8422 (ST-291) | 0.0487 |
| LPS vs. 231 (ST-17) | 0.0033 |  | 9427 (ST-144) vs. 104 (ST-1) | 0.008 |
| LPS vs. 9731 (ST-17) | 0.003 |  | 9427 (ST-144) vs. 123 (ST-498) | 0.0013 |
| LPS vs. 8422 (ST-291) | 0.0433 |  |  |  |

**Table S35: Statistically significant differences in *SLC2A1* gene expression 4 hours after macrophage infection with individual GBS isolates**, determined by one-way ANOVA with *post-hoc* Šidák’s test.

| **GBS isolates** | **Šidák test for multiple comparisons   (p-value)** |  | **GBS isolates** | **Šidák test for multiple comparisons   (p-value)** |
| --- | --- | --- | --- | --- |
| 229 (ST-23) vs. 7339 (ST-12) | 0.0220 |  | 203 (ST-8) vs. 123 (ST-498) | 0.0227 |
| 229 (ST-23) vs. 104 (ST-1) | 0.0404 |  | 231 (ST-17) vs. 7339 (ST-12) | 0.0131 |
| 229 (ST-23) vs. 123 (ST-498) | 0.0348 |  | 231 (ST-17) vs. 104 (ST-1) | 0.0200 |
| 203 (ST-8) vs. 7339 (ST-12) | 0.0143 |  | 231 (ST-17) vs. 123 (ST-498) | 0.0172 |
| 203 (ST-8) vs. 104 (ST-1) | 0.0265 |  |  |  |

**Table S36: Statistically significant differences in *SLC2A1* gene expression 24 hours after macrophage infection with individual GBS isolates**, determined by one-way ANOVA with *post-hoc* Šidák’s test.

| **GBS isolates** | **Šidák test for multiple comparisons   (p-value)** |  | **GBS isolates** | **Šidák test for multiple comparisons   (p-value)** |
| --- | --- | --- | --- | --- |
| LPS vs. 9427 (ST-144) | 0.0012 |  | 203 (ST-8) vs. 211 (ST-28) | 0.0085 |
| LPS vs. 203 (ST-8) | < 0.0001 |  | 203 (ST-8) vs. 7339 (ST-12) | 0.0028 |
| LPS vs. 211 (ST-28) | 0.0429 |  | 203 (ST-8) vs. 231 (ST-17) | 0.0007 |
| 229 (ST-23) vs. 203 (ST-8) | 0.0010 |  | 203 (ST-8) vs. 9731 (ST-17) | 0.0001 |
| 9427 (ST-144) vs. 6 (ST-1) | 0.0128 |  | 203 (ST-8) vs. 8422 (ST-291) | 0.001 |
| 9427 (ST-144) vs. 104 (ST-1) | 0.0328 |  | 203 (ST-8) vs. 6 (ST-1) | < 0.0001 |
| 9427 (ST-144) vs. 123 (ST-498) | 0.0102 |  | 203 (ST-8) vs. 104 (ST-1) | < 0.0001 |
| 203 (ST-8) vs. 10276 (ST-9) | 0.0002 |  | 203 (ST-8) vs. 123 (ST-498) | < 0.0001 |

**Table S37: Statistically significant differences in *PFKFB3* gene expression 4 hours after macrophage infection with individual GBS isolates**, determined by one-way ANOVA with *post-hoc* Šidák’s test.

| **GBS isolates** | **Šidák test for multiple comparisons   (p-value)** |  | **GBS isolates** | **Šidák test for multiple comparisons   (p-value)** |
| --- | --- | --- | --- | --- |
| LPS vs. 7339 (ST-12) | 0.0287 |  | 10276 (ST-9) vs. 8422 (ST-291) | 0.0307 |
| 10276 (ST-9) vs. 211 (ST-28) | 0.0081 |  | 211 (ST-28) vs. 9731 (ST-17) | 0.0260 |

**Table S38: Statistically significant differences in *PFKFB3* gene expression 24 hours after macrophage infection with individual GBS isolates**, determined by one-way ANOVA with *post-hoc* Šidák’s test.

| **GBS isolates** | **Šidák test for multiple comparisons   (p-value)** |  | **GBS isolates** | **Šidák test for multiple comparisons   (p-value)** |
| --- | --- | --- | --- | --- |
| LPS vs. 229 (ST-23) | 0.0052 |  | 7339 (ST-12) vs. 9731 (ST-17) | 0.0172 |
| LPS vs. 9427 (ST-144) | 0.0039 |  | 231 (ST-17) vs. 8422 (ST-291) | 0.0005 |
| LPS vs. 203 (ST-8) | 0.0038 |  | 231 (ST-17) vs. 6 (ST-1) | 0.0005 |
| LPS vs. 211 (ST-28) | 0.0235 |  | 231 (ST-17) vs. 104 (ST-1) | 0.0005 |
| LPS vs. 231 (ST-17) | < 0.0001 |  | 231 (ST-17) vs. 123 (ST-498) | 0.0005 |
| LPS vs. 9731 (ST-17) | < 0.0001 |  | 9731 (ST-17) vs. 8422 (ST-291) | 0.0047 |
| 10276 (ST-9) vs. 231 (ST-17) | < 0.0001 |  | 9731 (ST-17) vs. 6 (ST-1) | 0.0046 |
| 10276 (ST-9) vs. 9731 (ST-17) | 0.0004 |  | 9731 (ST-17) vs. 104 (ST-1) | 0.0053 |
| 211 (ST-28) vs. 231 (ST-17) | 0.0075 |  | 9731 (ST-17) vs. 123 (ST-498) | 0.005 |
| 7339 (ST-12) vs. 231 (ST-17) | 0.0015 |  |  |  |

**Table S39: Statistically significant differences in total caspase activity among macrophages stimulated with individual GBS isolates,** determined by one-way ANOVA with *post hoc* Šidák’s test.

| **GBS isolates** | **Šidák test for multiple comparisons   (p-value)** |  | **GBS isolates** | **Šidák test for multiple comparisons   (p-value)** |
| --- | --- | --- | --- | --- |
| 229 (ST-23) vs. 203 (ST-8) | 0.0191 |  | 10276 (ST-9) vs. 211 (ST-28) | 0.0002 |
| 229 (ST-23) vs. 231 (ST-17) | 0.0002 |  | 10276 (ST-9) vs. 7339 (ST-12) | 0.0006 |
| 229 (ST-23) vs. 9731 (ST-17) | 0.0027 |  | 10276 (ST-9) vs. 231 (ST-17) | <0.0001 |
| 229 (ST-23) vs. 6 (ST-1) | 0.0031 |  | 10276 (ST-9) vs. 9731 (ST-17) | <0.0001 |
| 229 (ST-23) vs. 104 (ST-1) | 0.003 |  | 10276 (ST-9) vs. 104 (ST-1) | <0.0001 |
| 9427 (ST-144) vs. 203 (ST-8) | 0.0275 |  | 10276 (ST-9) vs. 123 (ST-498) | <0.0001 |
| 9427 (ST-144) vs. 231 (ST-17) | <0.0001 |  | 211 (ST-28) vs. 6 (ST-1) | <0.0001 |
| 9427 (ST-144) vs. 9731 (ST-17) | 0.0014 |  | 7339 (ST-12) vs. 6 (ST-1) | <0.0001 |
| 9427 (ST-144) vs. 6 (ST-1) | 0.0047 |  | 231 (ST-17) vs. 8422 (ST-291) | <0.0001 |
| 9427 (ST-144) vs. 104 (ST-1) | 0.0016 |  | 231 (ST-17) vs. 6 (ST-1) | <0.0001 |
| 9427 (ST-144) vs. 123 (ST-498) | 0.0358 |  | 9731 (ST-17) vs. 8422 (ST-291) | 0.0004 |
| 203 (ST-8) vs. 211 (ST-28) | <0.0001 |  | 9731 (ST-17) vs. 6 (ST-1) | <0.0001 |
| 203 (ST-8) vs. 7339 (ST-12) | <0.0001 |  | 8422 (ST-291) vs. 6 (ST-1) | 0.002 |
| 203 (ST-8) vs. 231 (ST-17) | <0.0001 |  | 8422 (ST-291) vs. 104 (ST-1) | 0.0003 |
| 203 (ST-8) vs. 9731 (ST-17) | <0.0001 |  | 8422 (ST-291) vs. 123 (ST-498) | 0.0081 |
| 203 (ST-8) vs. 8422 (ST-291) | 0.0333 |  | 6 (ST-1) vs. 104 (ST-1) | <0.0001 |
| 203 (ST-8) vs. 104 (ST-1) | <0.0001 |  | 6 (ST-1) vs. 123 (ST-498) | <0.0001 |
| 203 (ST-8) vs. 123 (ST-498) | <0.0001 |  |  |  |

**Table S40: Statistically significant differences in specific caspase-1 activity among macrophages stimulated with individual GBS isolates,** determined by one-way ANOVA with *post hoc* Šidák’s test.

| **GBS isolates** | **Šidák test for multiple comparisons   (p-value)** |  | **GBS isolates** | **Šidák test for multiple comparisons   (p-value)** |
| --- | --- | --- | --- | --- |
| 229 (ST-23) vs. 203 (ST-8) | 0.0011 |  | 203 (ST-8) vs. 8422 (ST-291) | <0.0001 |
| 229 (ST-23) vs. 211 (ST-28) | 0.0298 |  | 203 (ST-8) vs. 104 (ST-1) | <0.0001 |
| 229 (ST-23) vs. 7339 (ST-12) | 0.029 |  | 203 (ST-8) vs. 123 (ST-498) | <0.0001 |
| 229 (ST-23) vs. 231 (ST-17) | <0.0001 |  | 10276 (ST-9) vs. 211 (ST-28) | 0.0002 |
| 229 (ST-23) vs. 9731 (ST-17) | <0.0001 |  | 10276 (ST-9) vs. 7339 (ST-12) | 0.0001 |
| 229 (ST-23) vs. 6 (ST-1) | 0.01 |  | 10276 (ST-9) vs. 231 (ST-17) | <0.0001 |
| 229 (ST-23) vs. 104 (ST-1) | <0.0001 |  | 10276 (ST-9) vs. 9731 (ST-17) | <0.0001 |
| 229 (ST-23) vs. 123 (ST-498) | 0.0066 |  | 10276 (ST-9) vs. 104 (ST-1) | <0.0001 |
| 9427 (ST-144) vs. 203 (ST-8) | 0.0059 |  | 10276 (ST-9) vs, 123 (ST-498) | <0.0001 |
| 9427 (ST-144) vs. 211 (ST-28) | 0.0053 |  | 211 (ST-28) vs. 6 (ST-1) | <0.0001 |
| 9427 (ST-144) vs. 7339 (ST-12) | 0.0052 |  | 7339 (ST-12) vs. 6 (ST-1) | <0.0001 |
| 9427 (ST-144) vs. 231 (ST-17) | <0.0001 |  | 231 (ST-17) vs. 8422 (ST-291) | <0.0001 |
| 9427 (ST-144) vs. 9731 (ST-17) | <0.0001 |  | 231 (ST-17) vs. 6 (ST-1) | <0.0001 |
| 9427 (ST-144) vs. 6 (ST-1) | 0.0418 |  | 9731 (ST-17) vs. 8422 (ST-291) | <0.0001 |
| 9427 (ST-144) vs. 104 (ST-1) | <0.0001 |  | 9731 (ST-17) vs. 6 (ST-1) | <0.0001 |
| 9427 (ST-144) vs. 123 (ST-498) | 0.001 |  | 8422 (ST-291) vs. 6 (ST-1) | 0.0004 |
| 203 (ST-8) vs. 211 (ST-28) | <0.0001 |  | 8422 (ST-291) vs. 104 (ST-1) | <0.0001 |
| 203 (ST-8) vs. 7339 (ST-12) | <0.0001 |  | 8422 (ST-291) vs. 123 (ST-498) | 0.0121 |
| 203 (ST-8) vs. 231 (ST-17) | <0.0001 |  | 6 (ST-1) vs. 104 (ST-1) | <0.0001 |
| 203 (ST-8) vs. 9731 (ST-17) | <0.0001 |  | 6 (ST-1) vs. 123 (ST-498) | <0.0001 |

**Table S41: Statistically significant differences in *CASP1* gene expression 24 hours after macrophage infection with individual GBS isolates**, determined by one-way ANOVA with *post-hoc* Šidák’s test.

| **GBS isolates** | **Šidák test for multiple comparisons   (p-value)** |  | **GBS isolates** | **Šidák test for multiple comparisons   (p-value)** |
| --- | --- | --- | --- | --- |
| 229 (ST-23) vs. 10276 (ST-9) | 0.0086 |  | 10276 (ST-9) vs. 123 (ST-498) | 0.0478 |
| 229 (ST-23) vs. 7339 (ST-12) | 0.0173 |  | 7339 (ST-12) vs. 231 (ST-17) | < 0.0001 |
| 203 (ST-8) vs. 231 (ST-17) | 0.0023 |  | 7339 (ST-12) vs. 9731 (ST-17) | 0.0075 |
| 10276 (ST-9) vs. 231 (ST-17) | < 0.0001 |  | 7339 (ST-12) vs. 104 (ST-1) | 0.0364 |
| 10276 (ST-9) vs. 9731 (ST-17) | 0.005 |  | 231 (ST-17) vs. 8422 (ST-291) | 0.0221 |
| 10276 (ST-9) vs. 104 (ST-1) | 0.0266 |  | 231 (ST-17) vs. 6 (ST-1) | 0.0011 |

**Table S42: Statistically significant differences in *CASP3* gene expression 4 hours after macrophage infection with individual GBS isolates**, determined by one-way ANOVA with *post-hoc* Šidák’s test.

| **GBS isolates** | **Šidák test for multiple comparisons   (p-value)** |  | **GBS isolates** | **Šidák test for multiple comparisons   (p-value)** |
| --- | --- | --- | --- | --- |
| 211 (ST-28) vs. 229 (ST-23) | 0.0118 |  | 211 (ST-28) vs. 6 (ST-1) | 0.001 |
| 211 (ST-28) vs. 9427 (ST-144) | 0.0196 |  | 211 (ST-28) vs. 123 (ST-498) | 0.0034 |
| 203 (ST-8) vs. 211 (ST-28) | 0.0013 |  | 7339 (ST-12) vs. 8422 (ST-291) | 0.0228 |
| 203 (ST-8) vs. 8422 (ST-291) | 0.0285 |  | 7339 (ST-12) vs. 104 (ST-1) | 0.0067 |
| 203 (ST-8) vs. 104 (ST-1) | 0.0085 |  | 231 (ST-17) vs. 104 (ST-1) | 0.0148 |
| 10276 (ST-9) vs. 211 (ST-28) | 0.0011 |  | 9731 (ST-17) vs. 8422 (ST-291) | 0.0308 |
| 10276 (ST-9) vs. 8422 (ST-291) | 0.0239 |  | 9731 (ST-17) vs. 104 (ST-1) | 0.0086 |
| 10276 (ST-9) vs. 104 (ST-1) | 0.0072 |  | 8422 (ST-291) vs. 6 (ST-1) | 0.0096 |
| 211 (ST-28) vs. 7339 (ST-12) | 0.0012 |  | 8422 (ST-291) vs. 123 (ST-498) | 0.0291 |
| 211 (ST-28) vs. 231 (ST-17) | 0.0038 |  | 104 (ST-1) vs. 6 (ST-1) | 0.0019 |
| 211 (ST-28) vs. 9731 (ST-17) | 0.0026 |  | 104 (ST-1) vs. 123 (ST-498) | 0.0027 |

**Table S43: Statistically significant differences in *CASP3* gene expression 24 hours after macrophage infection with individual GBS isolates**, determined by one-way ANOVA with *post-hoc* Šidák’s test.

| **GBS isolates** | **Šidák test for multiple comparisons   (p-value)** |  | **GBS isolates** | **Šidák test for multiple comparisons   (p-value)** |
| --- | --- | --- | --- | --- |
| 229 (ST-23) vs. 10276 (ST-9) | 0.0361 |  | 7339 (ST-12) vs. 231 (ST-17) | 0.0003 |
| 9427 (ST-144) vs. 231 (ST-17) | 0.0243 |  | 7339 (ST-12) vs. 9731 (ST-17) | 0.0109 |
| 203 (ST-8) vs. 231 (ST-17) | 0.0219 |  | 7339 (ST-12) vs. 104 (ST-1) | 0.0036 |
| 10276 (ST-9) vs. 211 (ST-28) | 0.0011 |  | 231 (ST-17) vs. 6 (ST-1) | 0.0002 |
| 10276 (ST-9) vs. 231 (ST-17) | < 0.0001 |  | 231 (ST-17) vs. 123 (ST-498) | 0.0075 |
| 10276 (ST-9) vs. 9731 (ST-17) | 0.0009 |  | 9731 (ST-17) vs. 6 (ST-1) | 0.0071 |
| 10276 (ST-9) vs. 8422 (ST-291) | 0.0053 |  | 8422 (ST-291) vs. 6 (ST-1) | 0.028 |
| 10276 (ST-9) vs. 104 (ST-1) | 0.0004 |  | 6 (ST-1) vs. 104 (ST-1) | 0.0012 |
| 211 (ST-28) vs. 7339 (ST-12) | 0.0147 |  | 104 (ST-1) vs. 123 (ST-498) | 0.0123 |
| 211 (ST-28) vs. 6 (ST-1) | 0.0143 |  |  |  |
